# Fast Isolation of Flavonoids from the Endemic Species *Nolana ramosissima* I.M. Johnst and Its Endothelium-Independent Relaxation Effect in Rat Aorta

**DOI:** 10.3390/molecules25030520

**Published:** 2020-01-24

**Authors:** Fredi Cifuentes, Javier Palacios, Jorge Bórquez, Adrián Paredes, Claudio Parra, Alejandra Bravo, Mario J. Simirgiotis

**Affiliations:** 1 Laboratorio de Fisiología Experimental (EPhyL), Instituto Antofagasta (IA), Universidad de Antofagasta, Casilla 170, Antofagasta 1271155, Chile; fredi.cifuentes@uantof.cl (F.C.); alejandrabravo.bq@gmail.com (A.B.); 2 Laboratorio de Bioquímica Aplicada, Instituto de EtnoFarmacología, Facultad de Ciencias de la Salud, Universidad Arturo Prat, Iquique 1110939, Chile; 3 Departamento de Química, Facultad de Ciencias Básicas, Universidad de Antofagasta, Casilla 170, Antofagasta 1271155, Chile; jorge.borquez@uantof.cl (J.B.); adrian.paredes@uantof.cl (A.P.); 4 Laboratorio de Productos Naturales y Química Medica, Facultad de Ciencias Agronómicas, Universidad de Tarapacá, Arica 1000000, Chile; cparra@cihde.cl; 5 Instituto de Farmacia, Facultad de Ciencias, Universidad Austral de Chile, Valdivia 5110566, Chile

**Keywords:** mass spectrometry, high-performance countercurrent chromatography, flavonoids, vasodilation, Chilean plants

## Abstract

The infusion of the desertic plant *Nolana ramosissima* I.M. Johnst showed vascular smooth muscle relaxation in rat aorta and the presence of several phenolic compounds, which were detected by high resolution UHPLC-Orbitrap-HESI-MS. In addition, five flavonoids were rapidly isolated from a methanolic extract using high-speed counter-current chromatography (HSCCC). The *N. ramosissima* extract showed endothelium-independent relaxation effect in rat aorta. Sixty-one compounds were detected in the infusion, mainly glycosylated flavonoids, flavanones and several oxylipins, suggesting that a synergistic effect between the compounds in the extracts could be responsible for the relaxation activity. Vascular activity experiments were done in isolated organ bath. In rat aorta, a nitric oxide inhibitor did not prevent the relaxation effects of the extract; however, a selective guanylyl cyclase inhibitor partially blunted this effect. The compound **5**,3′-dihydroxy-4′7-dimethoxyflavone presented higher relaxation effect than 100 μg/mL of *N. ramosissima* extract. The extract and the isolated metabolites from *N. ramosissima* can show relaxation effects on rat aorta by a mechanism that is independent of the endothelium.

## 1. Introduction

**Figure 1:**
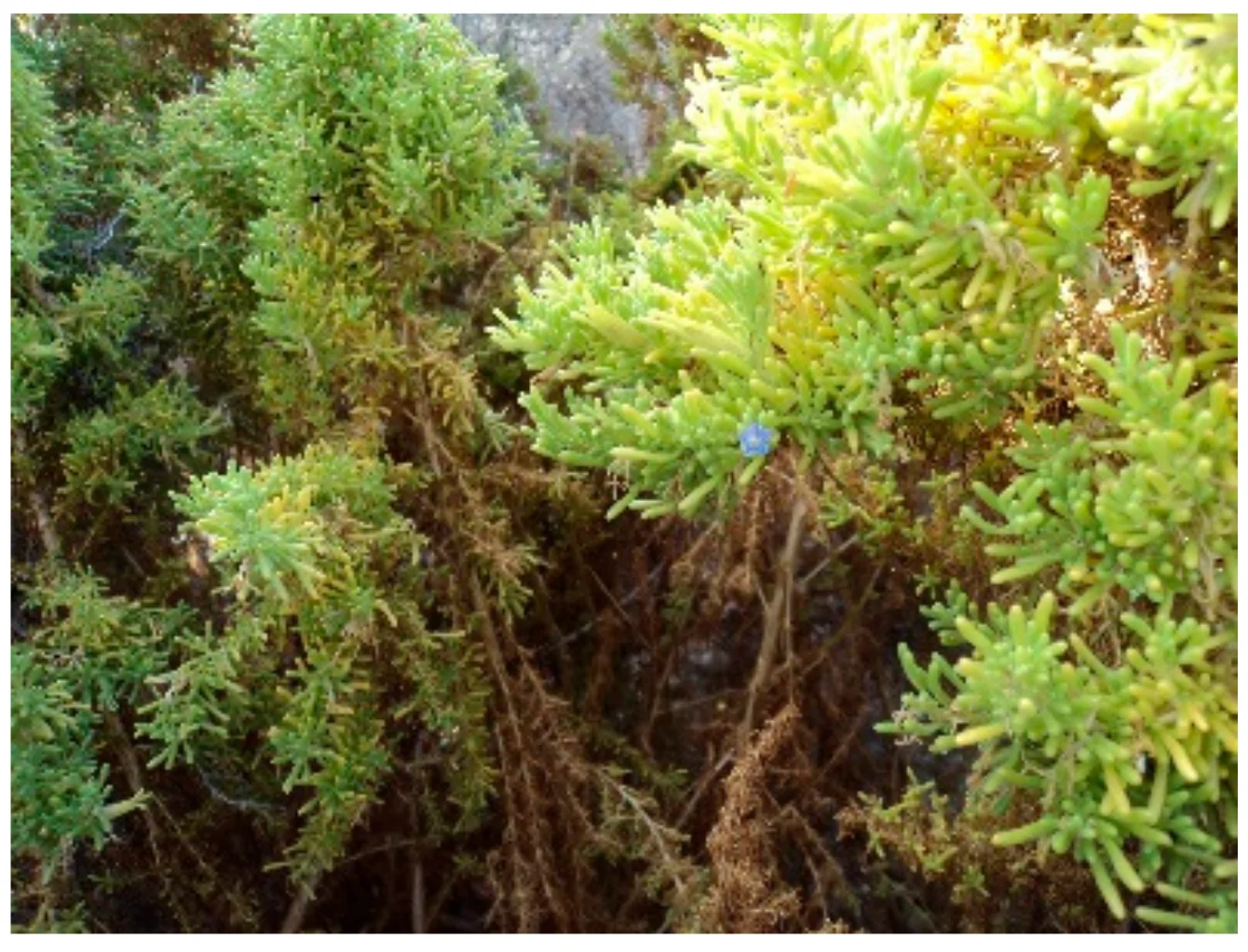
Picture of *N. ramosissima* I.M. Johnst. Collected in Paposo Valley, Atacama Desert, in October 2015.

*Nolana* species (*Solanaceae*) are one of the most important genera of the Atacama Desert represented by about 85 species and subspecies [1,2]. So far, a few species of *Nolana* were investigated regarding the chemical constituents by High-Resolution Liquid Chromatography-Mass Spectrometry (HPLC-MS) fingerprints. However, mostly labdane diterpenoids were reported until now in *Nolanas*. From *Nolana elegans* (Phil.) Reiche, new labdane diterpenoids were reported long time ago [3], and two labdanes were reported from *Nolana rostrate* (Lindl.) Miers ex Dunal [4] and two more labdanes were reported from *Nolana filifolia* I.M. Johnst [5]. In addition, from *Nolana coelestis* Miers ex Dunal four sesquiterpenoids were also reported [6]. N*olana sedifolia* Poepp. was shown to produce polyphenolics which are responsible for the antifungal activity against the fungus *Botrytis cinerea* [7]. We have reported few years ago the phenolic constituents and antioxidant activity of three *Nolanas* species, but the detection methodology employed was only Low Resolution Ion Trap-Mass Spectrometry (LR-ESI-MS) [8] and the study was incomplete, since we studied only ethyl acetate extracts. *N. ramosissima* is a species with blue bellflowers (Figure 1) which grows in the Chilean coastal area of Paposo valley at an altitude of 500–2000 m. High-speed counter-current chromatography (HSCCC) is a special liquid-liquid separation method which uses two immiscible phases, one is a stationary phase retained in a coil by a high centrifugal force, and the other is a mobile phase which is pumped through the stationary phase using an HPLC pump [9]. This methodology was broadly used to separate the flavonoids from plants and fruits [10,11,12,13]. HSCCC offer important advantages in separation of natural products: lower consumption of solvents, use of green chemistry solvents, such as water and ethyl acetate, no absorption on solid surfaces such as conventional column chromatography, very higher amounts of processing sample, introduction of crude extracts, and full recovery of natural products [14,15,16,17,18]. In this work we have applied this technique for the fast detection of flavonoids, from the methanolic extract of *N. ramosissima* for the testing of their relaxation activity in rat aorta. In addition, we discuss in this paper the relaxation activities of the polar extracts, (namely herbal tea or infusion and methanolic extract) of *N. ramosissima* plus their metabolite composition by UHPLC high-resolution orbitrap mass spectrometry. HPLC hyphenated with high resolution mass spectrometry with the help of diode array UV detection such as PDA Q-TOF-MS or PDA-HESI-Q-orbitrap HR-MS are outstanding techniques for the fast and accurate untargeted small metabolite analysis of plant samples [19,20]. This technique has been successfully used in the last few years by our group to analyze several endemic Chilean species [21,22,23,24].

## 2. Results and Discussion

### 2.1. Identification of the Compounds in N. ramosissima Methanol and Herbal Tea

**Figure 2:**
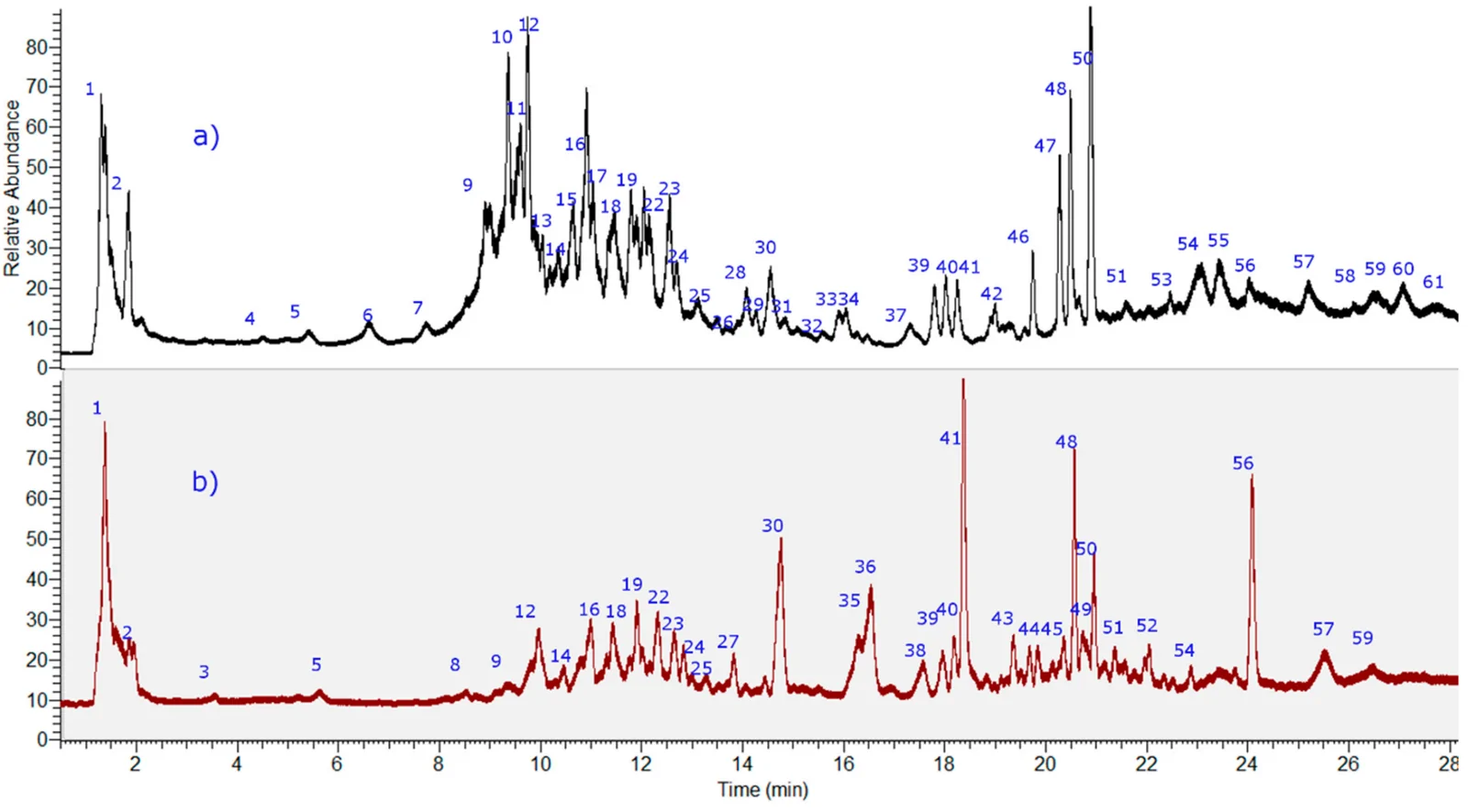
Photodiode array (PDA) chromatograms (UHPLC-PDA) of *N. ramosissima* extracts (**a**) methanol extract; (**b**) aqueous extract, at 280 nm.

Sixty-one compounds (56 in the methanolic extract and 42 in the infusion) were identified or tentatively identified by means of high resolution orbitrap mass spectrometry and PDA detection (Figure 2, Table S1 Supplementary Materials). The fast identification of the compounds is explained below.

#### 2.1.1. Flavonoids

**Figure 3:**
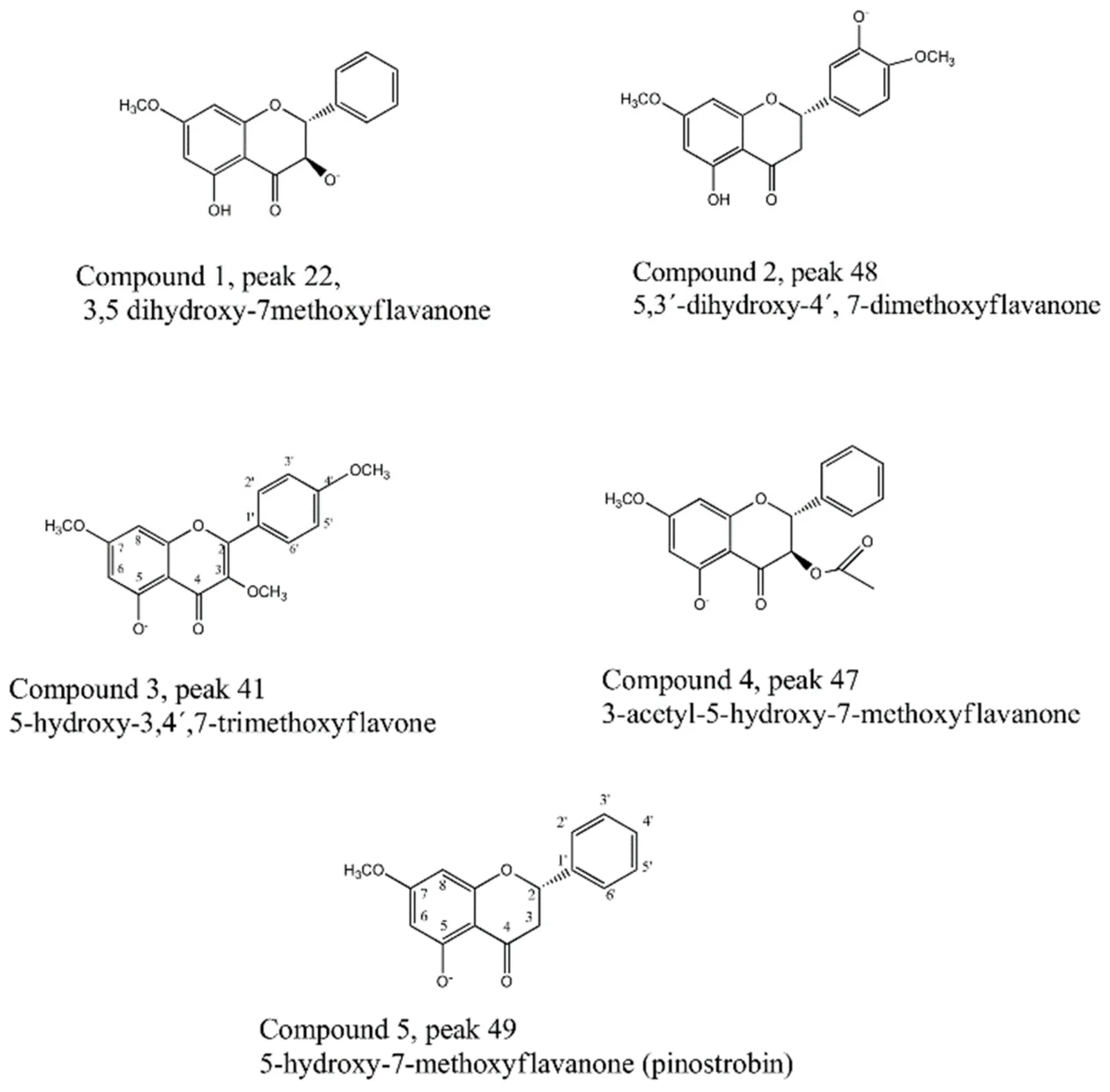
Structure of isolated flavonoids by high-speed counter-current chromatography (HSCCC).

**Figure 4:**
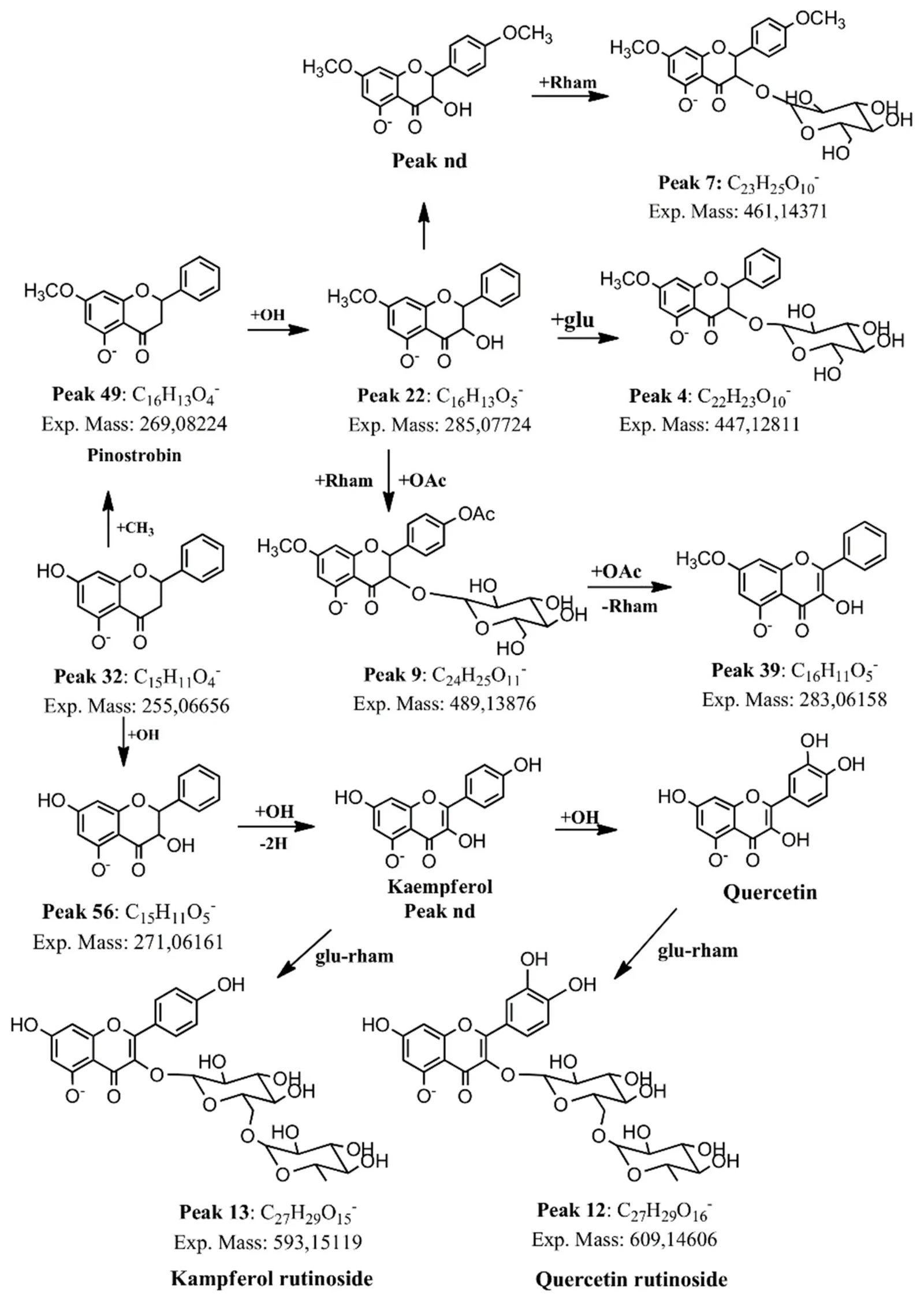
Biosynthetic relationship among flavonoids detected in *N. ramosissima*.

Several compounds were identified as flavanones (Figure 3 and Figure 4) and among them, some tentatively identified as naringenin derivatives [25]. Peak 7 with a [M − H]− ion at *m/z* 461.14371 was identified as the flavanone glycoside naringenin-4′,7-dimethoxyl-3-*O*-rhamnoside (C_23_H_25_O_10_^−^), while peak 9 with a pseudo-molecular ion at *m/z* 489.13876 was identified as the related glycosylated and acylated compound naringenin-4′-acetyl-7-methoxyl-3-*O*-rhamnoside (C_24_H_25_O_11_^−^), peak 15 with a [M − H]^−^ ion at *m/z* 517.17004 was identified as naringenin 3-hydroxyl-8-(3-methyl-2-butenyl)-7-*O*-glucoside (C_26_H_29_O_11_^−^) and peak 21 as the related naringenin derivative naringenin 3,4′-dimethoxyl-8-(3-methyl-2-butenyl) 7-O-glucoside ([M − H]^−^ ion at *m/z* 545.20111).

Peak 56 with a pseudomolecular ion at *m/z* 271.06161 was identified as naringenin by co-elution experiments with an authentic compound and peak 22 with a [M − H]^−^ ion at *m/z* 285.07687 as its O-methylated derivative 7-methoxynaringenin (C_16_H_13_O_5_^−^). Peak 19 ([M − H]^−^ ion at *m/z* 623.16132) was identified as isorhamnetin 3-O-rutinoside (C_28_H_31_O_16_^−^). Peak 17 with a [M − H]^−^ ion at *m/z* 531.18567 was identified as naringenin-3-hydroxyl-4′-methoxyl-8-*O*-(3-methyl-2-butenyl)-7-*O*-glucoside. Peak 11 with a [M − H]^−^ ion at *m/z* 503.15442 was identified as eriodictyol-5-acetyl-3′,4′-dimethoxyl-7-*O*-glucoside (C_25_H_27_O_11_^−^) and peak 4 as 7-methoxy-3-glucose-flavanone (C_22_H_23_O_10_^−^). Regarding flavonol compounds, peak 12 with a [M − H]^−^ ion at *m/z* 609.14606 was identified as rutin [25], identity confirmed using co-injection of the standard (C_27_H_29_O_16_^−^), while peak 13 with a [M − H]^−^ ion at *m/z* 593.15119 as kaempferol 3-*O*-rutinoside (C_27_H_29_O_15_^−^) [26] and peak 23 as quercetin-3-*O*-glucoside (C_27_H_29_O_16_^−^) [25]. Peak 39 with a [M − H]^−^ ion at *m/z* 283.06158 was identified as 7-methoxyapigenin (C_16_H_11_O_5_^−^) and peak 38 with a [M − H]^−^ ion at *m/z* 255.06653 was identified as 5,4′-dihydroxyflavanone, while peak 32 with a [M − H]^−^ ion at *m/z* 255.06656 was identified as its isomer 5,7-dihydroxyflavanone (C_15_H_11_O_4_^−^) and peak 40 as 5,7-dihydroxyflavone (C_15_H_9_O_4_^−^). Peak 49 with a [M − H]^−^ ion at *m/z* 269.08224 was identified as pinostrobin by using co-spiking experiments with authentic pinostrobin (C_16_H_13_O_4_^−^), peak 50 was identified as the aglycone apigenin (C_15_H_9_O_5_^−^) and peak 44 as quercetin (C_15_H_9_O_7_^−^). Peaks 30, 42 and 43 were identified as 7,3′-dimethoxyquercetin, 7,4′-dimethoxykaempferol and 7,4′-dimethoxyapigenin (C_17_H_13_O_7_^−^, C_17_H_13_O_6_^−^ and C_17_H_13_O_5_^−^), respectively.

#### 2.1.2. Fatty Acids

**Figure 5:**
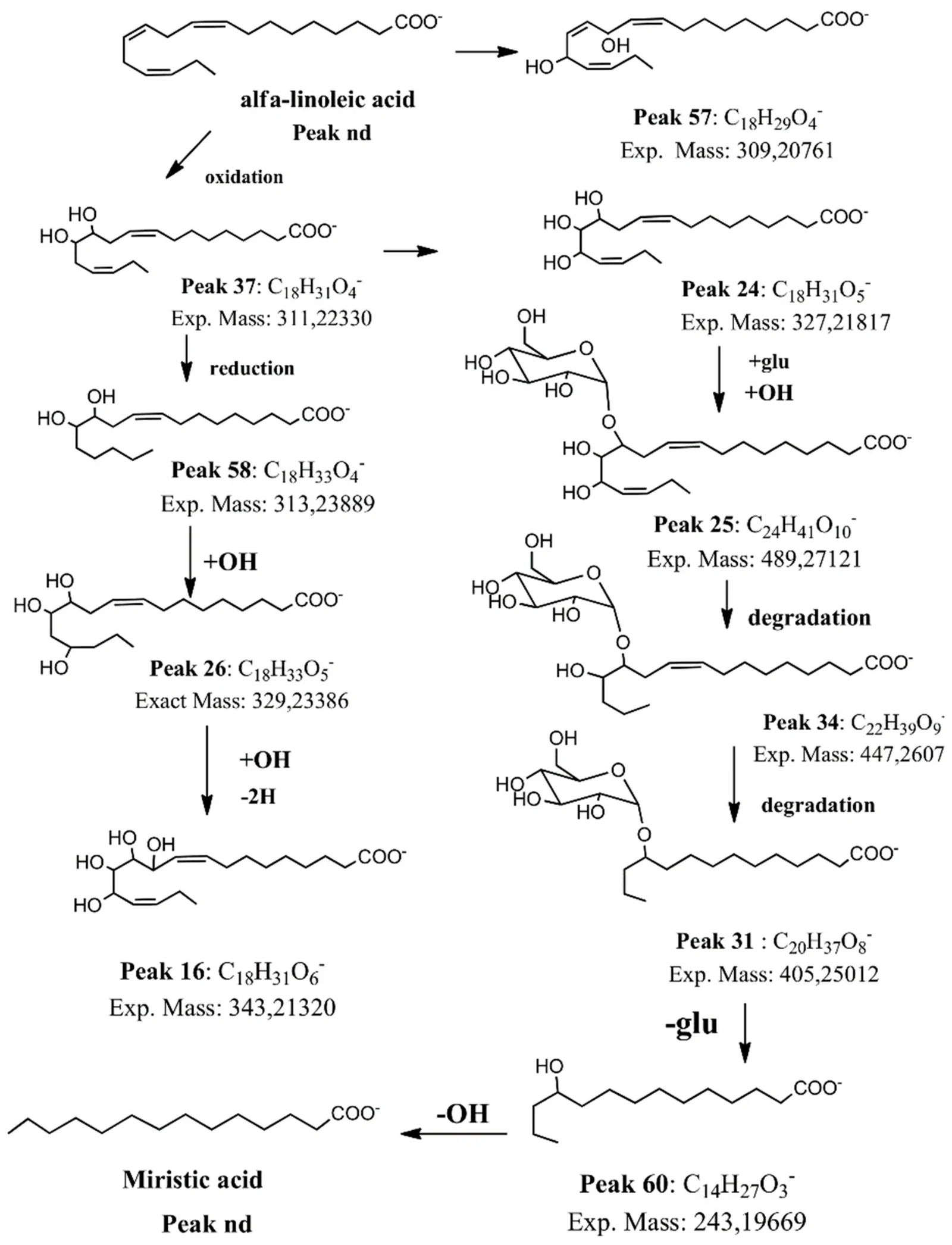
Biosynthetic relationship among fatty acids detected in *N. ramosissima*.

Several compounds were identified the important dietary antioxidant fatty acids known as oxylipins [26,27] (Figure 5). 

Thus, Peak 51 with a [M − H]^−^ ion at *m/z* 315.25458 was identified as dihydroxy-octadecanoic acid (C_18_H_35_O_4_^−^); peak 45 with a [M − H]^−^ ion at *m/z* 355.32230 was identified as hydroxy-docosanoic acid (C_22_H_43_O_3_^−^) and peak 20 as 10-hydroxy-6-oxodecanoic acid (C_10_H_17_O_4_^−^). We could detect also some glycosylated fatty acids; thus, peak 25 with a [M − H]^−^ ion at *m/z* 489.27121 was identified as dihydroxy-octadecadienoic acid–O-glucoside (C_24_H_41_O_10_^−^), while peak 34 with a [M − H]^−^ ion at *m/z* 447.2599 was identified as hydroxy-hexadecadienoic acid-*O*-glucoside (C_22_H_39_O_9_^−^). Peak 37 with a [M − H]^−^ ion at *m/z* 311.22330 was identified as dihydroxy-octadecadienoic acid (C_18_H_31_O_4_^−^) [26], peak 58 with a [M − H]^−^ ion at *m/z* 313.23889 as 9,10-dihydroxy-octadecenoic acid (C_18_H_33_O_4_^−^) [26] and peak 53 as tetrahydroxy-eicosadienoic acid (C_20_H_35_O_6_^−^). In a similar manner, Peak 20 (ion at *m/z* 201.11302) was identified as 10-hydroxy-6-oxodecanoic acid (C_10_H_17_O_4_^−^), and peak 24 (ion at *m/z* 327.2181) was identified as trihydroxy-octadecadienoic acid (C_18_H_31_O_5_^−^). In the same way, peak 25 was identified as the glycosyl derivative dihydroxy-octadecadienoic acid-*O*-glucoside (C_24_H_41_O_10_^−^) and peak 33 as hydroxy-eicosaenoic acid glucoside (ion at *m/z* 503.32269). Peaks 28 and 27 were identified as 9,10-dihydroxy-hexadecanoic acid and 9,10-tetradecanoic acid (C_16_H_31_O_4_^−^ and C_14_H_27_O_4_^−^), respectively, while peak 17 with a [M − H]− ion at *m/z* 217.10805 was identified as 9,10-dihydroxy-6-oxodecanoic acid (C_10_H_17_O_5_^−^) [26], and peak 16 with a [M − H]^−^ ion at *m/z* 343.21320 was identified as trihydroxy-octadecadienoic acid (C_18_H_31_O_5_^−^). Peak 59 with a [M − H]^−^ ion at *m/z* 369.30176 was identified as dihydroxydocosenoic acid (C_22_H_41_O_4_^−^) while isomer peaks 52 and 55 were identified as tetrahydroxy-eicosatrienoic acids (C_20_H_33_O_6_^−^). Peak 26 with a [M − H]^−^ ion at *m/z* 329.23386 was identified as trihydroxy-octadecaenoic acid (C_18_H_33_O_5_^−^) and peak 29 as its isomer (C_18_H_33_O_5_^−^) as previously reported by us to occur in the mesocarp of Keule fruits [27]. Peak 24 with 2 a.m.u of difference (327.21817) was identified as trihydroxy-octadecadienoic acid (C_18_H_31_O_5_^−^) [27]. Peak 57 with a [M − H]^−^ ion at *m/z* 309.20761 was identified as dihydroxy-octadecatrienoic acid (C_18_H_29_O_4_^−^), in contrast to a compound detected in Asparagus, which was identified as 15-hydroperoxy-octadecatrienoic acid [26]. Peak 33 with a [M − H]^−^ ion at *m/z* 517.17004 was identified as hydroxy-hexadecaenoic acid-*O*-glucoside (C_22_H_39_O_9_^−^). The saturated fatty acids Hydroxypalmitic (C_16_H_31_O_3_^−^, peak 61), hydroxymiristic acid (C_14_H_27_O_3_^−^, peak 60) and its glucoside derivative miristic acid-11-*O*-glucoside (C_20_H_37_O_8_^−^, peak 31) were also detected (Table S1, Supplementary Materials). Peak 1 with a [M − H]^−^ ion at *m/z* 209.06636 was identified as 2,4,5,6,7-pentahydroxypentanoic acid (C_7_H_13_O_7_^−^).

#### 2.1.3. Coumarins

Peak 18 with a [M − H]^−^ ion at *m/z* 191.03490 was identified as the simple coumarin scopoletin (C_10_H_7_O_4_^−^)[27]. Peak 3 with a molecular ion at *m/z* 339.07178 was identified as the glucoside coumarin derivative esculetin-6-*O*-glucoside (sculin C_15_H_15_O_9_^−^) [28], while peak 2 with a [M − H]^−^ ion at *m/z* 377.08542 was identified as sculetin-5-hydroxy-7-methoxy-6-*O*-glucoside (C_18_H_17_O_9_^−^), and peak 6 with a molecular ion at *m/z* 369.08267 was identified as 5-hydroxy-7-methoxyesculin (C_16_H_17_O_10_^−^).

#### 2.1.4. Phenolic Acids

Peak 5 with [M − H]^−^ ion at *m/z* 153.01881 was identified as 2,4-dihydroxybenzoic acid (C_7_H_5_O_4_^−^) [29], peak 8 as salicylic acid (C_7_H_5_O_3_^−^) and peak 10 as free caffeic acid (C_9_H_7_O_4_^−^) [30].

### 2.2. Fast HSCCC Isolation of Major Compounds in N. ramosissima Methanol Extract

The employment of immiscible solvent systems in our HSCCC machine allowed the fast isolation of the main five components (compounds **1**–**5**) from a crude methanol extract of *N. ramosissima*. Major isolated compounds **1**–**5** were identified by their ESI-MS, GC-MS data and mono and bidimentional NMR spectra. (Please see Supplementary Materials, Figures S4–S32). Furthermore, from the extrusion partition of the HSCCC run, only hydroxypalmitic acid (Peak 61) and inseparable mixtures of fatty acids were isolated. Other potential approaches to pre-treat and extract the active compounds are nowadays employed, including experimental approaches for the identification after elimination of the effect of matrix on quantitative analyses by HPLC−MS, such as CO_2_ extraction, pretreatment with ammonia and hydrogen peroxide, pressurized solvents; etc., however, infusion is the typical edible form, and methanol extraction at ambient temperature proved to be good solvent to extract all the flavonoids and phenolics in our *Nolana* species for purification purposes [31,32,33].

Compound **1**: 3,5-dihydroxy-7-methoxyflavanone (peak 22). Colourless crystals, m.p. 178.8–179.0 °C. [M − H]^−^: 285.0765, MS^2^: 267 [M − H_2_O]^−^, 251 [M − H_2_O − CH_3_]^−^. ^1^H NMR (300 MHz, CDCl_3_) δ ppm: 3.65 (s, OCH_3_), 4.59 (1H, d, *J* = 12 Hz), 5.10 (1H, d, *J* = 12 Hz), 6.10 (1H, d, *J* = 17.3 Hz), 6.12 (1H, d, *J* = 17,3 Hz), 7.52 (3H, m), 7.69 (2H, m), 11.24 (1H, s, OH). ^13^C NMR (300 MHz, CDCl_3_) δ ppm: 56.60 (OCH_3_), 72.21 (C-2), 82.61 (C-3), 194.95 (C-4), 162.46 (C-5), 96.07 (C-6), 168.85 (C-7), 93.82 (C-8), 163.46 (C-9), 100.55 (C-10), 136.01 (C-1′), 127.56 (C-2′), 128.89 (C-3′), 127.56 (C-4′), 128.59 (C-5′), 127.56 (C-6′). These data are in agreement with the literature [34,35,36].

Compound **2**: 5,3′-dihydroxy-4′, 7-dimethoxyflavanone (Peak 48). Colourless crystals, m.p. 123.0–125.0 °C [M − H]^−^: 315.0874, MS^n^: 283.8 [M – H − CH_3_]^−^, 254.7 [M – H − CH_3_ − CO]^−^. ^1^H NMR (300 MHz, CDCl_3_) δ ppm: 2.65 (1H, dd, *J* = 3.1 and 16.4Hz), 3.05 (1H, dd, *J* = 16.4 and 12.5 Hz), 3.75 (s, OCH_3_), 3.80 (s, OCH_3_), 5.54 (1H, dd, *J* = 12.5 and 3.1 Hz), 6.21 (1H, d, *J* = 2,3 Hz), 6.23 (1H, d, *J* = 2,3 Hz), 7.4 (1H, m), 7.5 (2H, m), 11.24 (1H, s, OH).^13^C NMR (300 MHz, CDCl_3_) δ ppm: 56.32 (OCH_3_), 56.20 (OCH_3_), 78.63 (C-2), 45.23 (C-3), 188.07 (C-4), 164.68 (C-5), 94.17 (C-6), 165.89 (C-7), 93.35 (C-8), 162.11 (C-9), 105.83 (C-10), 139.40 (C-1′), 126.96 (C-2′), 128.90 (C-3′), 162.00 (C-4′), 128.90 (C-5′), 126.96 (C-6′). These data are in agreement with the literature [35].

Compound **3**: 5-hydroxy-3,4′,7-trimethoxyflavone (peak 41). Yellow crystals, m.p. 148.0–149.0 °C. [M − H]^−^: 327.0874, MS^n^: 312 [M – H − CH_3_]^−^, 314 [M + H − CH_3_]^+^, 297 [M – H − 2CH_3_]^-^, 299 [M + H − 2CH_3_]^+^. ^1^H NMR (300 MHz, CDCl_3_) δ ppm: 3.87 (s, OCH_3_), 3.89 (s, OCH_3_) 3.92 (s, OCH_3_), 6.38 (1H, d, *J* = 2,3 Hz), 6.47 (1H, d, *J* = 2,3 Hz), 7.04 (1H, d, *J* = 9.2 Hz), 8.10 (1H, d, *J* = 9.2 Hz), 11.24 (1H, s, OH).^13^C NMR (300 MHz, CDCl_3_) δ ppm: 55.10 (OCH_3_), 56.02 (OCH_3_), 60.49 (OCH_3_), 155.0 (C-2), 122.9 (C-3), 194.15 (C-4), 161.70 (C-5), 98.31 (C-6), 164.88 (C-7), 92.91 (C-8), 161.22 (C-9), 105.45 (C-10), 138.27 (C-1′), 114.06 (C-2′), 129.30 (C-3′), 157.62 (C-4′), 129.30 (C-5′), 114.06 (C-6′). These data are in agreement with the literature [35,37,38]. 

Compound **4**: 3-acetyl-5-hydroxy-7-methoxyflavanone (peak 47). Colourless crystals, m.p. 96–97 °C [M − H]^−^: 327.0874. MS^n^: 268.9 [M + H − CH_3_COOH]^+^, 255.0 [M + H − CH_3_COOH − CH_3_]^+^. ^1^H NMR (300 MHz, CDCl_3_) δ ppm: 2.05 (s, OCOCH_3_), 3.83 (s, OCH_3_), 5.39 (1H, d, *J* = 11.8 Hz), 5.84 (1H, d, *J* = 11.8 Hz), 6.09 (1H, d, *J* = 2,3 Hz), 6.14 (1H, d, *J* = 2,3 Hz), 7.45 (5H, m),11.51 (1H, s, OH).^13^C NMR (300 MHz, CDCl_3_) δ ppm: 20.33 (OCOCH_3_), 55.87 (OCH_3_), 81.36 (C-2), 72.42 (C-3), 191.70 (C-4), 164.13 (C-5), 95.69 (C-6), 169.27 (C-7), 94.73 (C-8), 168.53 (C-9), 101.88 (C-10), 135.16 (C-1′), 128.91 (C-2′), 129.59 (C-3′), 127.38 (C-4′), 129.59 (C-5′), 128.91 (C-6′). These data are in agreement with the literature [39].

Compound **5**: 5-hydroxy-7-methoxy-flavanone (peak 49), Pinostrobin. Colourless crystals, m.p. 119.7–120.0 °C. [M − H]^−^: 269.0818, MS^2^: 241.9, 178.3, 161.8, 153.2. ^1^H NMR (300 MHz, CDCl_3_) δ ppm: 2.84 (1H, dd, *J* = 17.2 and 3.1 Hz), 3.11 (1H, dd, *J* = 13.0 and 17.2 Hz), 3.80 (s, OCH_3_), 5.44 (1H, dd, *J* = 3.1 and 13. Hz), 6.10 (1H, d, *J* = 2.3 Hz), 6.09 (1H, d, *J* = 2,3 Hz), 7.47 (5H, m), 12.11 (1H, s, OH). ^13^C NMR (300 MHz, CDCl_3_) δ ppm: 55.91 (OCH_3_), 79.14 (C-2), 42.75 (C-3), 196.16 (C-4), 162.44 (C-5), 94.62 (C-6), 167.94 (C-7), 94.13 (C-8), 163.87 (C-9), 102.77 (C-10), 137.86 (C-1’), 126.96 (C-2′), 129.16 (C-3’), 126.96 (C-4′), 129.16 (C-5’), 126.96 (C-6′). These data are in agreement with the literature [35,40]. Furthermore the X-ray crystal structure of this compound was already published by us [41].

### 2.3. N. ramosissima Induced Relaxation in Aortic Ring of Rat, Endothelium-Independent Activity

**Figure 6:**
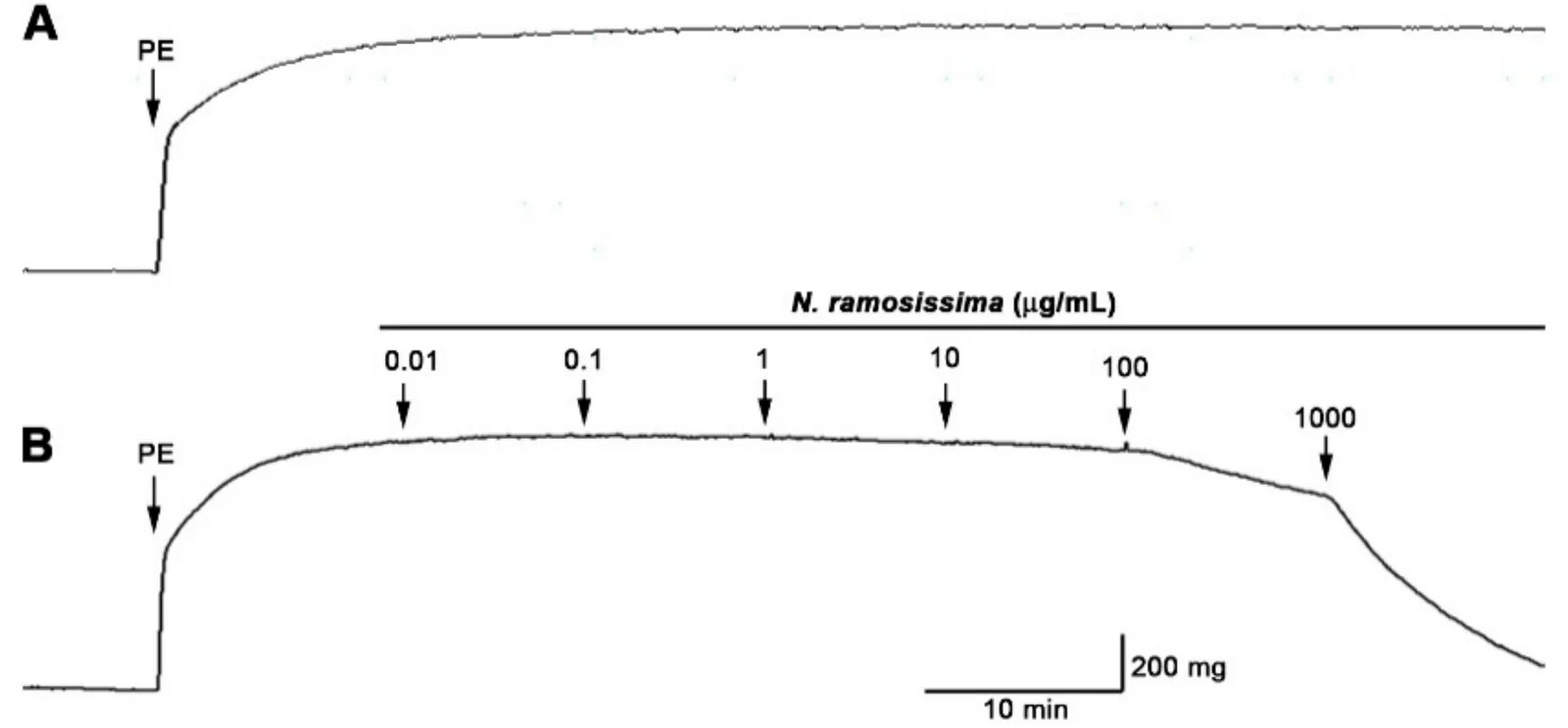
Original record of the relaxation effects of *N. ramosissima* in intact rat aorta. Rat aorta was pre-contracted with 10^−^^6^ M phenylephrine (PE) for 10 min, and then, cumulative concentrations of *N. ramosissima* (0.01 to 1000 g/mL) were added in organ bath at 7 min intervals (**B**) to compare with the control (**A**).

We found that *N. ramosissima* could have a potential antihypertensive effect, since it caused a relaxation effect on rat aortic rings pre-contracted with PE (Figure 6). 

**Figure 7:**
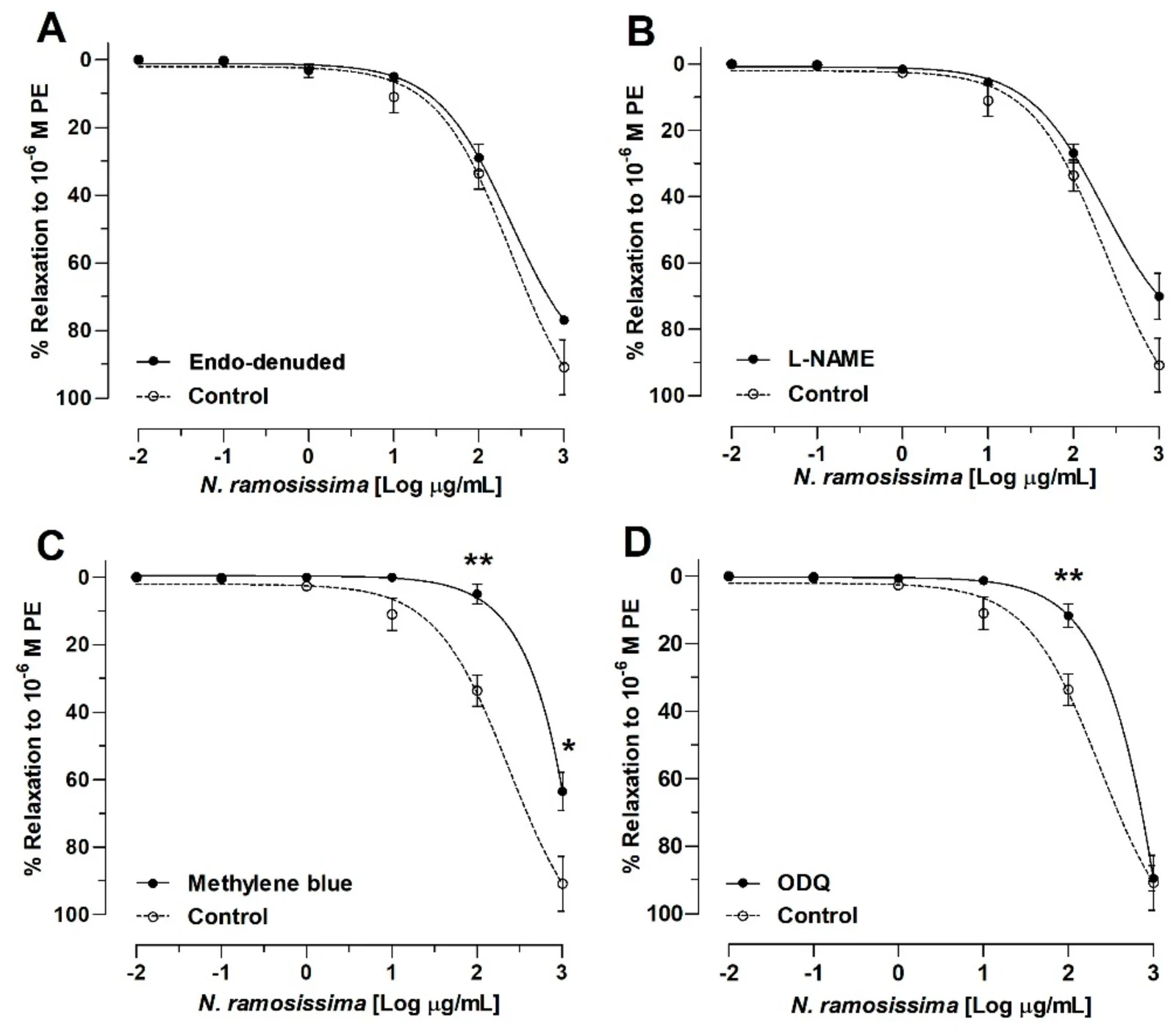
Relaxation effect of *N. ramosissima* in rat aorta. Arteries were pre-contracted with 10^−6^ M PE. Concentration-response curves for *N. ramosissima* extract in intact and denuded-endothelium aortic rings (**A**) in absence (Control) or in presence of 10^−4^ M L-NAME (**B**), 10^−6^ M methylene blue (**C**) or 10^−6^ M 1H-(1,2,4) oxadiazolo [4 ,3-a]quinoxalin-1-one (ODQ) (**D**) in rat aorta. Methylene blue and ODQ are non-selective and selective inhibitors of soluble guanylyl cyclase, respectively. Data are the average ± standard error of the mean (SEM) of 5 independent experiments. *p < 0.05, **p < 0.01 vs. Control.

*N. ramosissima* produced the concentration-dependent relaxation in intact aortic rings (34 ± 5% with 2 [log µg/mL] or 100 µg/mL versus 91 ± 8% with 3 [log µg/mL] or 1000 µg/mL; *p* < 0.001) and denuded (29 ± 4% with 2 [log µg/mL] or 100 µg/mL versus 77 ± 1% with 3 [log µg/mL] or 1000 µg/mL; *p* < 0.001; Figure 7A). 

Although relaxation effect observed with *N. ramosissima* did not involve endothelial nitric oxide synthase, the soluble guanylate cyclase pathway it was (Figure 7). The pre-incubation with an inhibitor of nitric oxide synthase (10^−4^ M, N(ω)-nitro-L-arginine methyl ester (L-NAME) did not reduce the relaxation to *N. ramosissima* in intact aorta (Figure 7B). 

However, compared with control (34 ± 5% with 2 [log µg/mL] or 100 µg/mL), the pre-incubation with methylene blue, a nonspecific soluble guanylyl cyclase inhibitor, significantly decreased the relaxation in intact aorta (5 ± 3% with 2 [log µg/mL] or 100 µg/mL; *p* < 0.01; Figure 7c). Methylene blue has been used extensively to inhibit soluble guanylyl cyclase, the effector that mediates the vasodilator effect of nitric oxide [42]. The pre-incubation with 10^-6^ M 1H-(1,2,4) oxadiazolo[4,3-a]quinoxalin-1-one (an inhibitor of soluble guanylyl cyclase; ODQ) reduced the relaxation and confirmed that soluble guanylyl cyclase is involved on vascular relaxation by the extract (12 ± 4% with 2 [log µg/mL] or 100 µg/mL; *p* < 0.01; Figure 7D). The log half-maximal inhibitor concentration (Log IC_50_) of ODQ was significantly (*p* < 0.05) different in the presence of extract versus control (Table 1).

These findings suggest that *N. ramosissima* produced relaxation in intact and endothelium-denuded rat aorta when they were exposed to cumulative concentrations of the extract. Commonly, vasodilators substances induced the activation of soluble guanylyl cyclase, but a few can stimulate nitric oxide-independent soluble guanylate cyclase activity and thus produce relaxation [43,44,45]. Thus, it is possible that *N. ramosissima* caused relaxation of vascular smooth muscle by directly stimulating the nitric oxide-independent soluble guanylate cyclase (sGC) and cGMP pathway [46].

### 2.4. N. ramosissima Reduced the Contractile Response to KCl and Phenylephrine

To study whether the effect of *N. ramosissima* on vascular response is mediated by the membrane depolarization or pharmacological stimulation, the contractile response to KCl and PE was evaluated.

**Figure 8:**
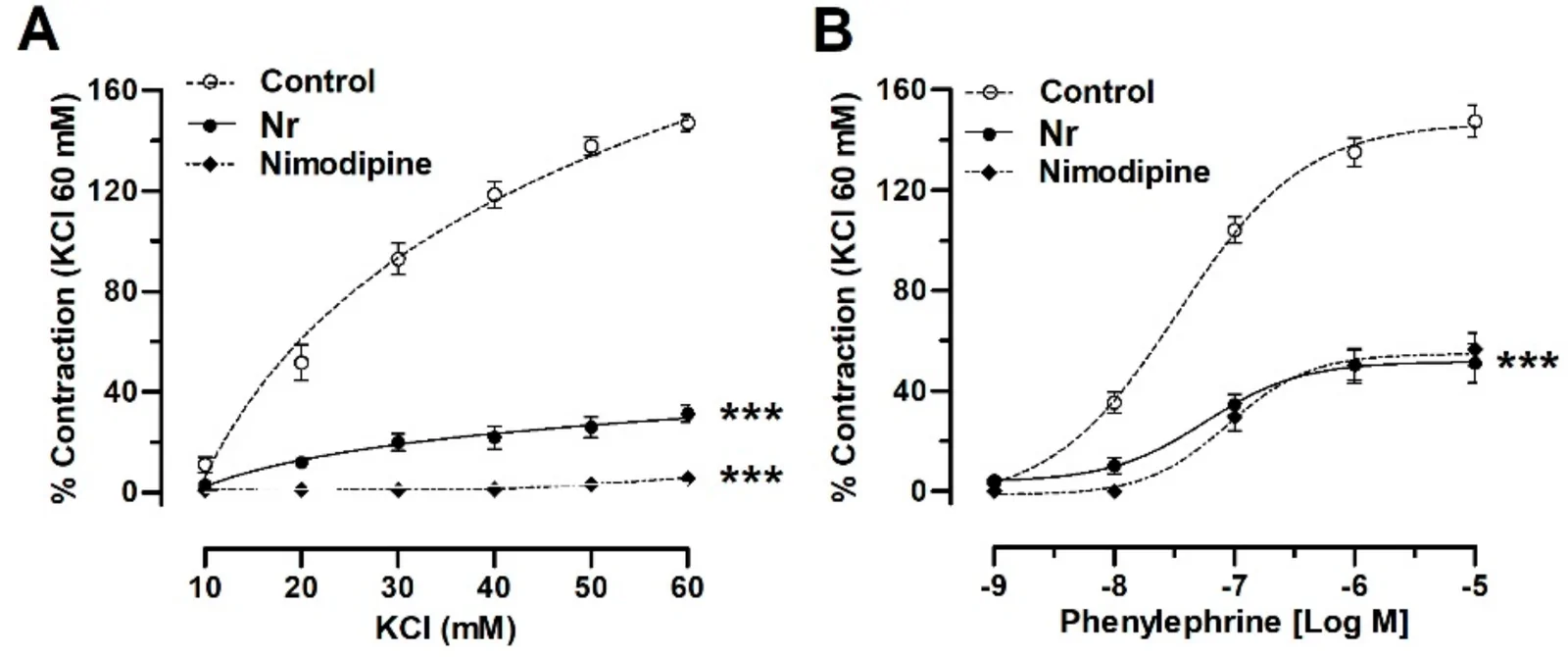
*N. ramosissima* decreases the vascular contractile response to KCl and PE. The vascular tissue was pre-incubated in absence (control) or presence of 100 μg/mL of extract or nimodipine (10^-6^ M) for 20 min before adding KCl (10–60 mM) (**A**) or PE (10^−9^ − 10^−5^ M) (**B**). Data are the average ± SEM of 5 independent experiments. Statistically significant differences: *** *p* < 0.001 vs control.

The pre-incubation with the extract significantly reduced the maximal contractile response to KCl (144 ± 4% control vs. 31 ± 3%; *p* < 0.001; Figure 8A) and to PE (139 ± 8% control vs. 75 ± 8%; *p* < 0.001; Figure 8B). The log half-maximal effective concentration (Log EC_50_) to KCl and PE was not significantly different in the presence of extract versus control, indicating that the extract did not modify the sensitivity of K^+^ channels or alpha-adrenergic receptor (Table 2).

Interestingly, the blockage of L-type voltage-gated Ca^2+^ channels (Cav1.2) with 10^-6^ M nimodipine decreased the contractile response to KCl (5 ± 2%) and PE (57 ± 7%) in a similar way than *N. ramosissima* (Figure 8B). Therefore, this comparison with nimodipine suggests that extract-induced vascular effect is major by blocking of Ca^2+^ influx through the plasmatic membrane [24].

### 2.5. Pure Compounds of N. ramosissima Induced Relaxation

**Figure 9:**
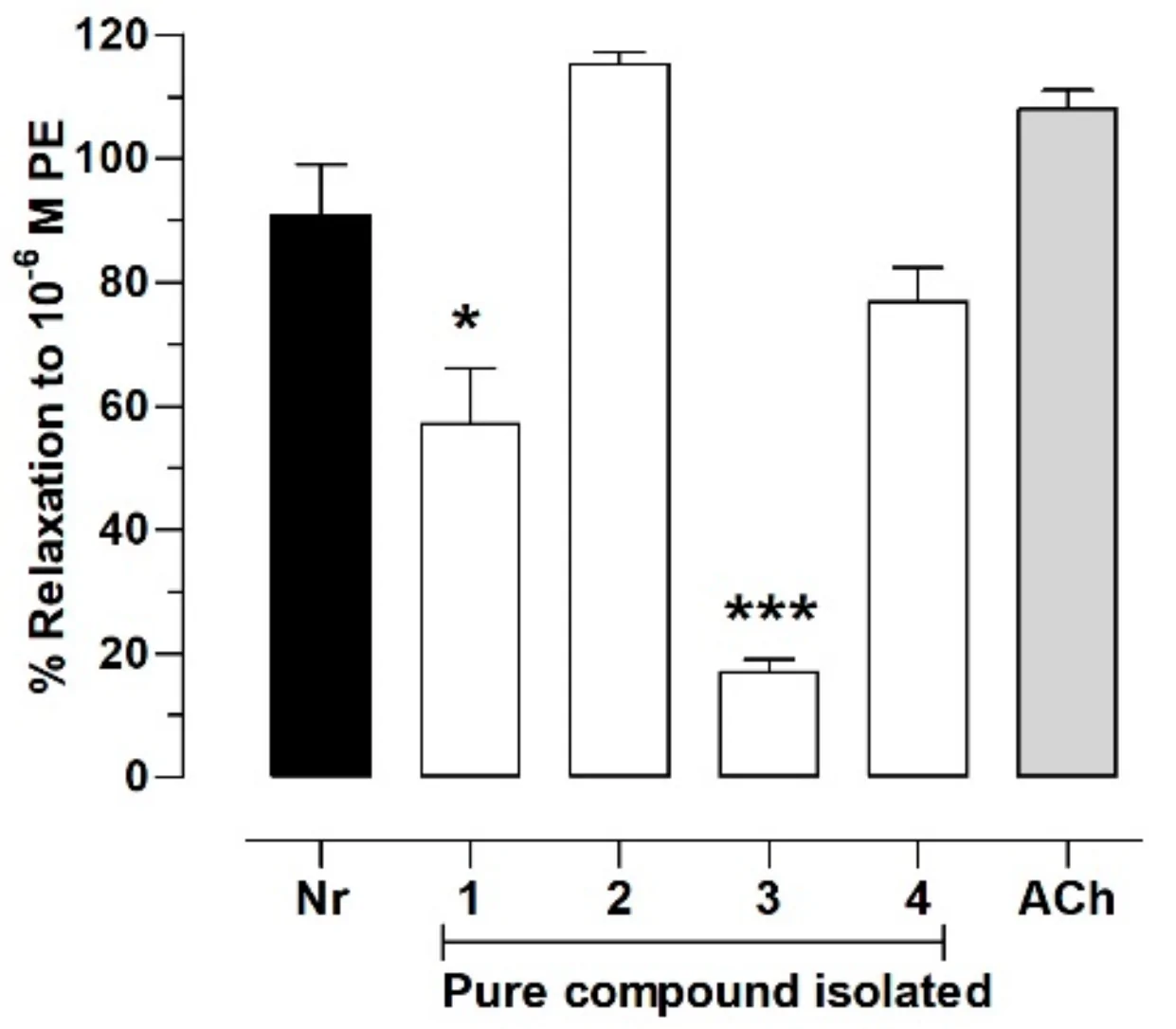
Screening test of 4 pure compounds of *N. ramosissima* on vascular response in rat aorta. Relaxation effect of extract 100 μg/mL *N. ramosissima* (black bar; Nr), 4 pure compounds isolated (1–4; 10^−4^ M) and acetylcholine (ACh; 10^−4^ M) in intact aortic rings pre-constricted with 10^−6^ M PE. The compound 2 and 4 presented a similar relaxation than 100 μg/mL *N. ramosissima* extract. Values are mean ± standard error of the mean of 4 experiments. Statistically significant differences: *p < 0.05, ***p < 0.001 vs. Nr.

Isolated compounds **1**–**4** showed different vascular relaxation in rat aorta pre-contracted with 10^-6^ M PE. The relaxation effect was compared with the extract of *N. ramosissima* and an agonist dependent drug on endothelial nitric oxide, acetylcholine. As shown in Figure 9, only the isolated compound **2** (115 ± 2%; 10^−^^4^ M) and 4 (77 ± 5%; 10^−^^4^ M) possessed an important relaxation effect in intact aortic rings. Interestingly, compound **2** presented a higher relaxation than 100 μg/mL *N. ramosissima* extract (91 ± 8%). Compound **2** is 5,3′-dihydroxy-4′7-dimethoxyflavone and compound **4** is 3-acetyl-5-hydroxy-7-methoxyflavone. Apparently the free OH groups in position 5 and 3′ is important for the increase of this activity.

## 3. Materials and Methods

### 3.1. Chemicals

HPLC-MS solvents and Gradient Grade (GR) acetonitrile, methanol, hexane and ethyl acetate were from Merck (Santiago, Chile). Ultrapure water was obtained from a Millipore water purification system (Milli-Q Merck Millipore, Chile). HPLC standards, quercetin, isorhamnetin, kaempferol, naringenin, eriodictyol, hesperetin, rhamnetin, linoleic acid, (all standards with purity higher than 95% by HPLC) were purchased either from Extrasynthèse (Genay, France), Sigma Aldrich (Saint Louis, Missouri, MO, USA) or ChromaDex (Santa Ana, California, CA, USA). TLC: Silica gel 60 F_254_ plates (Merck, Darmstadt, Germany). Column chromatography: Sephadex LH-20, MeOH as solvent. A Quattro *semi preparative* MK-7 *HSCCC* instrument (AECS inc., Bridgend, UK) with a total capacity of 437 mL (Figure S1, Supplementary Materials) with two bobbins, each of them bearing two stainless steel coils (one bobbin with two coils, an analytical of 27 mL, 1 mm tubing bore, one preparative of 205 mL and 2.1 mm i.d. and the other bobbin bearing two 116 mL, 2.1 mm tubing bore preparative coils). The mobile phase pumped using two Series II SSI model HPLC pumps (LabAlliance, Pennsyivania, PA, USA) and fractions collected with a Gilson FC 203B model fraction collector (Middleton, MI, USA). The effluent was monitored using a UV–visible-ECOM Flash 06 S single 254 nm wavelength detector governed by Ecomac software (Ecom, Prague, Czech Republic). Nuclear Magnetic Resonance (NMR) spectroscopy: ^1^H-, and ^13^C- and 2D NMR spectra: Bruker Avance 400 or Bruker Avance II 600 UltraShield spectrometers: δ in ppm relative to Me_4_Si as internal standard, *J* in Hz. The melting point was measured in a Stuart Scientific apparatus SMP3 (Bibby, London UK). L-phenylephrine hydrochloride (PE), acetylcholine chloride (ACh), 1H-(1,2,4)oxadiazolo[4,3-a]quinoxalin-1-one (ODQ), N^ω^-nitro-L-arginine methyl ester (L-NAME) were purchased to Sigma-Aldrich (St Luis, MO, USA). Nimodipine was obtained from Merck (Darmstadt, Germany). Several drugs were dissolved in distilled deionized water (deionized water Millipore) and kept at 4 °C. The stock solution of ODQ and nimodipine was dissolved in dimethyl sulfoxide (DMSO; 0.1% final concentration) (Merck, Germany). The extract of *N. ramosissima* was dissolved in physiological Krebs–Ringer bicarbonate buffer (KRB) in all vascular experiments.

### 3.2. Plant Material

*N. ramosissima* was collected in Paposo Valley, northern Chile in April 2011 and was identified by the botanist Alicia Marticorena (University of Concepción, Chile). A voucher specimen is deposited at the Natural Products’ laboratory, University of Antofagasta, Chile, with the number Nr-111004-1.

### 3.3. Extraction

Approximately 100 g of the dried plant was pulverized in a mortar and then extracted with 500 mL of HPLC-MS grade methanol in the dark in an ultrasonic bath for one hour (three times); the extracts were combined, filtered and evaporated in vacuo in the dark (40 °C) to give 7.83 g of *N. ramosissima* methanolic extract. For the preparation of the herbal tea, 2 g of the pulverized plant was added distilled water (250 mL) at 45 °C and left to stand for 12 h. The plant material was then filtered and the solution lyophilized to give 0.63 g of lyophilized material.

### 3.4. UHPLC-PDA-MS Instrument

For UHPLC Photodiode-Array–Mass-Spectrometry (UHPLC-PDA-MS) analysis, 5 mg of the methanol extract and lyophilized herbal tea (infusion) were individually dissolved in 2 mL of methanol; filtered (using a PTFE 200 m filter) and 10 L were injected in the instrument. A Thermo Scientific Ultimate 3000 UHPLC system equipped with a quaternary Series RS pump and a Thermo Scientific Dionex Ultimate 3000 Series TCC-3000RS column compartments with Ultimate 3000 Series WPS-3000RS autosampler and a rapid separations photodiode array (PDA) detector controlled by Chromeleon 7.2 Software (Thermo Fisher Scientific, Darmstadt, Germany) hyphenated with a Thermo high resolution Q-Exactive focus mass spectrometer (Thermo, Bremen, Germany) were used for analysis. The chromatographic system was coupled to the MS with a Heated Electrospray Ionization Source II (HESI II). Nitrogen (purity > 99.999%) obtained from a Genius NM32LA nitrogen generator (Peak Scientific, Billerica, Massachusetts, MA, USA) was employed as both the collision and damping gas. Mass calibration for Orbitrap was performed once a day, in both (−) and (+) modes, to ensure a working mass accuracy lowers than or equal to 5 ppm. N-butylamine and Cafeine, (Sigma Aldrich, Saint Louis, Missouri, Mo, USA) were the calibration standards for positive ions and taurocholic acid sodium salt, buspirone hydrochloride, sodium dodecyl sulfate, (Sigma Aldrich, Saint Louis, Missouri, MO, USA) were used as negative standards to calibrate the spectrometer. These compounds were added to a mixture of acetonitrile, acetic acid, water, and methanol (Merck Darmstadt, Germany) and afterwards infused using a Chemyx Fusion 100 syringe pump (Thermo Fisher Scientific, Bremen, Germany). XCalibur 2.3 and Trace Finder 3.2 software (Thermo Fisher Scientific, San José, California, CA, USA) were used for UHPLC control and data processing, respectively. Q Exactive 2.0 SP 2 from Thermo Fisher Scientific was used to control the mass spectrometer.

### 3.5. LC Parameters

The separations were done using an UHPLC C18 column (Acclaim, 150 mm × 4.6 mm ID, 2.5 m, Thermo Fisher Scientific, Bremen, Germany) at 25 °C. The detection wavelengths were 254, 280 and 320 nm, and PDA from 200 to 800 nm was recorded. Mobile phases were 1% formic aqueous solution (A) and acetonitrile (B). The gradient program (time (min), % B) was: (0.00, 7); (5.00, 7); (10.00, 35); (15.00, 40); (20.00, 70); (25.00, 70); (35.00, 7) and 12 min for column equilibration before each injection. The flow rate was 1.00 mL min^−1^, and the injection volume was 10 L. Standards and extracts dissolved in methanol were kept at 10 °C during storage in the autosampler.

### 3.6. MS Parameters

The HESI II parameters were sheath gas flow rate, 75 units; aux. gas unit flow rate, 20; capillary temperature, 400 °C; aux gas heater temperature, 500 °C; spray voltage, 2500 V (for ESI-); and S lens RF level, 30. Full scan data in negative mode was acquired at a resolving power of 70,000 full width half maximum (FWHM) at *m/z* 200. For the compounds of interest, a scan range of *m/z* 100–1000 was chosen; the automatic gain control (AGC) was set at 3e^6^, and the injection time was set to 200 ms. Scan-rate was set at 2 scans s^−1^. External calibration was performed using a calibration solution in positive and negative modes before each sample series. In addition to the full scan acquisition method, for confirmations purposes, a targeted MS/MS analysis was performed using the mass inclusion list and expected retention times of the target analytes, with a 30 s time window, with the Orbitrap spectrometer operating both in positive and negative mode at 17,500 FWHM (*m/z* 200). The AGC target was set to 2e^5^, with the maximum injection time of 20 ms. The precursor ions filtered by the quadrupole operates at an isolation window of *m/z* 2. The fore vacuum, high vacuum and ultrahigh vacuum were maintained at approximately 2 mbar, 10^5^ and below 10^10^ mbar, respectively. Collision energy (HCD cell) operated at 30 kv. Detection was based on calculated exact mass and on retention time of target compounds. The mass tolerance window was set to 5 ppm.

### 3.7. Selection of the Solvent System for HSCCC

According to the requirements for solvent systems in HSCCC [47], the selection was performed by a partition experiment of the crude extract using several solvent systems including (1) hexane:acetonitrile (stationary phase poorly retained in the coil), (2) hexane: methanol, (3) HEMWAT (n-hexane: ethyl acetate: methanol: water) and (4) n-hexane: ethanol: water at different volume ratios. The measurement of *K* values of target flavonoids from crude sample was as follows: A portion of the crude methanol extract (2 mg) was weighed into a 5 mL glass tube and added 1 mL of each phase of a pre-equilibrated two-phase solvent system. The glass tube was capped and then placed in a vortex mixer for 5 min to equilibrate the sample between the two-phases. After settling, the two phases were separated and evaporated to dryness. The residues were diluted with 1 mL methanol and 20 μL of the resulting solution was injected into the UHPLC system. Then, the quantitative UHPLC-PDA was performed by UHPLC. The *K* value was expressed as the peak area of target compounds in the upper phase (stationary phase) divided by that in the lower phase (mobile phase). The best liquid–liquid separation system, in our opinion, for the UV active compounds from *N. ramosissima* petroleum ether extract was the biphasic non-aqueous solvent system: n-hexane: ethanol: water 6:5:1 v/v/v (Table S2, Supplementary Materials).

### 3.8. HSCCC Separation of N. ramosissima Methanol Extract

After equilibration of the two solvents in a separating funnel, the two resulting phase layers were separated shortly before use and degassed in an ultrasonic bath (for 15 min). The upper phase was then used as stationary phase and the lower phase as mobile phase in the ‘*head-to-tail*’ mode. The separation was performed using temperature control during the separation (approx. 25 °C) with a rotation velocity of 800 rpm. The columns of the *HSCCC* were then filled with upper phase, and the lower mobile phase was pumped at a flow rate of 5.0 mL/min using the ‘*head-to-tail*’ mode. After the mobile phase front emerged and the hydrodynamic equilibrium was established in the columns, the percentage of the retention of the stationary phase (75%) was recorded. Then the dried methanol extract of *N. ramosissima* (500 mg) was dissolved in 5 mL each of upper and lower phase, filtered through a 0.45 m micropore membrane (PTFE, Waters), introduced via a plastic syringe to a 10 mL sample loop and then directly injected into the separation column through a manual low-pressure sample injection valve (Rheodyne, Cotati, CA, USA). For recovery of all existing *N. ramosissima* metabolites, a two column volume with *elution* and *extrusion* steps was applied [48]. The effluent from the outlet of the column was collected (10 mL/tube, 5 mL/min), and 32 fractions were collected in the elution mode (numbered F1–F32). Then, the system was changed to the *extrusion* mode with pumping of stationary phase at a lower spinning velocity (400 rpm) and the same flow rate. Every 2 min the *extrusion*-fractions were collected (F33 until F 50). Component detection of the effluent was performed with UV-light (λ = 254 nm, Figure S2, Supplementary Materials), and visualization of the spots in a TLC plate (Figure S3, Supplementary Materials) of each collected tube (Silica gel F^254^, Merck Darmstadt, Germany, developed with n-hexane:Ethyl acetate 8:2, *v*/*v*) with the universal spray reagent p-anisaldehyde-concentrated sulphuric acid-glacial acid (1:2:97, *v*/*v*/*v*), and flash heating (110 °C) on a hot plate [11].

### 3.9. Isolation and Identification of Compounds

The HSCCC fractions I–IV (Figures S2 and S3, Supplementary Materials) collected in the elution mode were refined by Gel-permeation chromatography on Sephadex LH-20 (5 cm x 25 cm, 100 g, eluted with HPLC grade methanol) to yield flavonoids 1–5 (peaks 22, 48, 41, 47 and 49). From fraction I: tubes 12–14 (96–112 mL), 32 mg of compound **1** were obtained, from fraction II: tubes 15–16 (120–128 mL), 15.3 mg of compound **2**, were obtained, from fraction III: tubes 17–20 (130–160 mL), 18 mg of compound **3** and 12 mg of compound **4** were obtained and from fraction IV: tubes 21–26 (168–208 mL), 73 mg of compound **5** were isolated. From the extrusion fractions, (tubes 32–40) (Figure S3, Supplementary Materials) inseparable mixtures of fatty acids could be detected by TLC analysis.

### 3.10. Animals

For vascular reactivity experiments, female Sprague–Dawley rats (6–8 weeks of age, 170–200 g) from the breeding colony at the Antofagasta University were used. All animals were housed in a temperature-controlled, light-cycled (08:00–20:00 h) room with *ad libitum* access to drinking water and standard rat chow (Champion, Santiago). The investigation conformed to the Guide for the Care and Use of Laboratory Animals published by the U. S. National Institutes of Health (NIH Publication revised 2013), and the local animal research committee approved the experimental procedure used in the present study (number 135/2018).

### 3.11. Isolation of Aortic Rings

The procedure of these experiments was realized in accordance to described by Cifuentes et al. [24]. Rats were sacrificed through cervical dislocation. The thoracic aorta was quickly excised and placed in physiological Krebs-Ringer bicarbonate buffer (KRB) containing (mM): 4.2 KCl, 1.19 KH_2_PO_4_, 120 NaCl, 25 NaHCO_3_, 1.2 MgSO_4_, 1.3 CaCl_2_, and 5 d-glucose (pH 7.4). Rings (3-5 mm and 2-4 mg) were prepared after connective tissue was cleaned out from the aorta, taking special care to avoid endothelial damage. Aortic rings were equilibrated for 40 min in KRB at 37 °C by constant bubbling with 95% O_2_ and 5% CO_2_.

### 3.12. Vascular Reactivity Experiments

Aortic rings from the same animal were studied in duplicate, using different vasoactive substances (phenylephrine [PE], KCl and acetylcholine [ACh]). The rings were mounted on two 25-gauge stainless steel wires; the lower one was attached to a stationary glass rod and the upper one was attached to an isometric transducer (Radnoti, Monrovia, California, CA, USA). The transducer was connected to a PowerLab 8/35 (Colorado Springs, Colorado, CO, USA) for continuous recording of vascular tension using the LabChart Pro v8.1.2 computer program (ADInstrument). After the equilibration period for 40 min, the aortic rings were stabilized by 3 successive near-maximum contractions with KCl (60 mM) for 10 min. The passive tension on aorta was 1.0 g, which was determined to be the resting tension for obtaining maximum active tension induced by 60 mM KCl. To study the effect of methanolic extract or pure compounds on vascular reactivity in rat aorta, we performed different protocols. In the first protocol, the aortic rings were pre-contracted with 10^−6^ M PE, and then increasing concentrations of *N. ramosissima* or pure compounds were added to the bath. In the second protocol, the rat aorta was pre-incubated in presence of *N. ramossima* for 20 min, followed by a contraction with 10^−6^ M PE. A stock solution in DMSO (10^−3^ M) was prepared with pure compounds, and then, dilutions in KRB were added in the bath. In some experiments, the endothelium removal was by gently rubbing it off using a small piece of cotton. To evaluate the vascular function of the endothelium, the relaxation to 10^−6^ M acetylcholine (muscarinic agonist) in pre-contracted aortic rings with 10^−6^ M PE was tested. According to the general use of rat aorta as a pharmacological tool for in vitro, the aortic rings were considered with a functional endothelial response if relaxation was up to 70% to 80% [49].

## 4. Conclusions

Five major flavones were quickly isolated by HSCCC from a methanol extract of the endemic species *N. ramosissima*, and four of them showed relaxation activity. Besides, some 61 compounds were detected in both *N. ramosissima* polar extracts by UHPLC-MS. Of those, four were coumarins (peaks 2, 3, 6 and 18), 15 flavanones (peaks 4, 7, 9, 11, 15, 17, 21, 22, 32, 38, 46–49 and 56), 12 flavones (peaks 12, 13, 19, 23, 30, 39, 40–44 and 50), 3 phenolic acids (peaks 5,8 and 10) and 22 oxylipins/fatty acids (peaks 1, 14, 16, 20, 24, 29, 31, 33–37, 51-55, 57–61). Compound **2** presented higher relaxation effects than *N. ramosissima* extract. Moreover, since the methanolic extract and infusion of the plant showed higher relaxation effect than the isolated compounds (**1**, **3**, **4**); it can be assumed that these compounds present in the extract could have a synergistic effect and boost the hypotensive or antihypertensive activity. Furthermore, *N. ramosissima* caused relaxation through an endothelium-independent mechanism; this effect could be exerted by pure compounds **2** and **4**. Regarding these results, *N. ramosissima* could be used as a natural medicine to lower blood pressure. However, more research is needed to support the use of this plant as an antihypertensive agent.

## Figures and Tables

**Table 1:** Effect of *N. ramosissima* (Nr) on the vascular response to different vasoactive substances on the nitric oxide pathway in rat aorta.

Drugs	Log (IC_50_) (g/mL)
Control	2.38 ± 0.12
Endo-denuded	2.37 ± 0.06
L-NAME	2.35 ± 0.10
Methylene blue	
ODQ	3.51 ± 0.38 *

Vasoactive substances: 10^−4^ M L-NAME, 10^−6^ M methylene blue and 10^−6^ M 1H-(1,2,4) oxadiazolo[4,3-a]quinoxalin-1-one (ODQ). Log (IC_50_) represent the logarithm half-maximal inhibitory concentration. The values are mean ± standard error of the mean (S.E.M.) and n = 5. Statistically significant difference * *p* < 0.05 vs. Control.

**Table 2:** Effect of *N. ramosissima* (Nr.; 100 μg/mL) on the contractile response to KCl (10–60 mM), and phenylephrine (PE; 10^-9^ to 10^-5^ M) in intact rat aorta.

Drugs	Log (EC_50_)
KCl (mM)	
Control	1.57 ± 0.15
Nr	1.63 ± 0.50
Nimodipine	
PE (nM) Control	−7.47 ± 0.10
Nr	−7.18 ± 0.26
Nimodipine	−7.05 ± 0.14

Log (EC_50_) represents the logarithm of half-maximal effective concentration of drug. The values are mean ± SEM, representing the mean of 5 independent experiments.

## Data Availability

The datasets generated during and/or analyzed during the current study are available from the corresponding author on reasonable request.

## Supplementary Materials

The following are available online.
