# Correction: Cifuentes et al. Fast Isolation of Flavonoids from the Endemic Species *Nolana ramosissima* I.M. Johnst and Its Endothelium-Independent Relaxation Effect in Rat Aorta. *Molecules* 2020, *25*, 520

**DOI:** 10.3390/molecules31152651

**Published:** 2026-07-30

**Authors:** Fredi Cifuentes, Javier Palacios, Jorge Bórquez, Adrián Paredes, Claudio Parra, Alejandra Bravo, Mario J. Simirgiotis

**Affiliations:** 1 Laboratorio de Fisiología Experimental (EPhyL), Instituto Antofagasta (IA), Universidad de Antofagasta, Casilla 170, Antofagasta 1271155, Chile; fredi.cifuentes@uantof.cl (F.C.); alejandrabravo.bq@gmail.com (A.B.); 2 Laboratorio de Bioquímica Aplicada, Instituto de EtnoFarmacología, Facultad de Ciencias de la Salud, Universidad Arturo Prat, Iquique 1110939, Chile; 3 Departamento de Química, Facultad de Ciencias Básicas, Universidad de Antofagasta, Casilla 170, Antofagasta 1271155, Chile; jorge.borquez@uantof.cl (J.B.); adrian.paredes@uantof.cl (A.P.); 4 Laboratorio de Productos Naturales y Química Medica, Facultad de Ciencias Agronómicas, Universidad de Tarapacá, Arica 1000000, Chile; cparra@cihde.cl; 5 Instituto de Farmacia, Facultad de Ciencias, Universidad Austral de Chile, Valdivia 5110566, Chile

In the original publication [1] high-performance counter-current chromatography (HPCCC) was replaced by high-speed counter-current chromatography (HSCCC).

**Error in Figure Caption**

In the original publication, there was a mistake in Figure 3’s caption as published. High-performance counter-current chromatography (HPCCC) was used instead of high-speed counter-current chromatography (HSCCC). The corrected Figure 3 caption appears below.

**Text Correction**

There was an error in the original publication in Section 2.2, title: 2.2. Fast HPCCC Isolation of Major Compounds in *N. ramosissima* Methanol Extract.

A correction has been made to 2.2. Fast HSCCC Isolation of Major Compounds in *N. ramosissima* Methanol Extract.

In the paragraph of Section 2.2, HPCCC was replaced with HSCCC:

“The employment of immiscible solvent systems in our HSCCC machine allowed the fast isolation of the main five components (compounds **1**–**5**) from a crude methanol extract of *N. ramosissima*.”

There was an error in the original publication in Section 3.7. *Selection of the Solvent System for HPCCC*.

A correction has been made to *3.7. Selection of the Solvent System for HSCCC*.

In the paragraph of Section 3.7, HPCCC was replaced with HSCCC:

“According to the requirements for solvent systems in HSCCC [47], the selection was performed by a partition experiment of the crude extract using several solvent systems including (1) hexane:acetonitrile (stationary phase poorly retained in the coil), (2) hexane: methanol, (3) HEMWAT (n-hexane: ethyl acetate: methanol: water) and (4) n-hexane: ethanol: water at different volume ratios.”

**References**

With this correction, References 31, 32 and 33 have been updated:

31. Azmir, J.; Zaidul, I.S.M.; Rahman, M.M.; Sharif, K.M.; Mohamed, A.; Sahena, F.; Jahurul, M.H.A.; Ghafoor, K.; Norulaini, N.A.N.; Omar, A.K.M. Techniques for extraction of bioactive compounds from plant materials: A review. *J. Food Eng.* **2013**, *117*, 426–436.
32. Zhang, Q.-W.; Lin, L.-G.; Ye, W.-C. Techniques for extraction and isolation of natural products: A comprehensive review. *Chin. Med.* **2018**, *13*, 20. https://doi.org/10.1186/s13020-018-0177-x.
33. Zakaria, S.M.; Kamal, S.M.M. Subcritical Water Extraction of Bioactive Compounds from Plants and Algae: Applications in Pharmaceutical and Food Ingredients. *Food Eng. Rev.* **2015**, *8*, 23–34.

The authors state that the scientific conclusions are unaffected. This correction was approved by the Academic Editor. The original publication has also been updated.

## Figures and Tables

**Figure 3:**
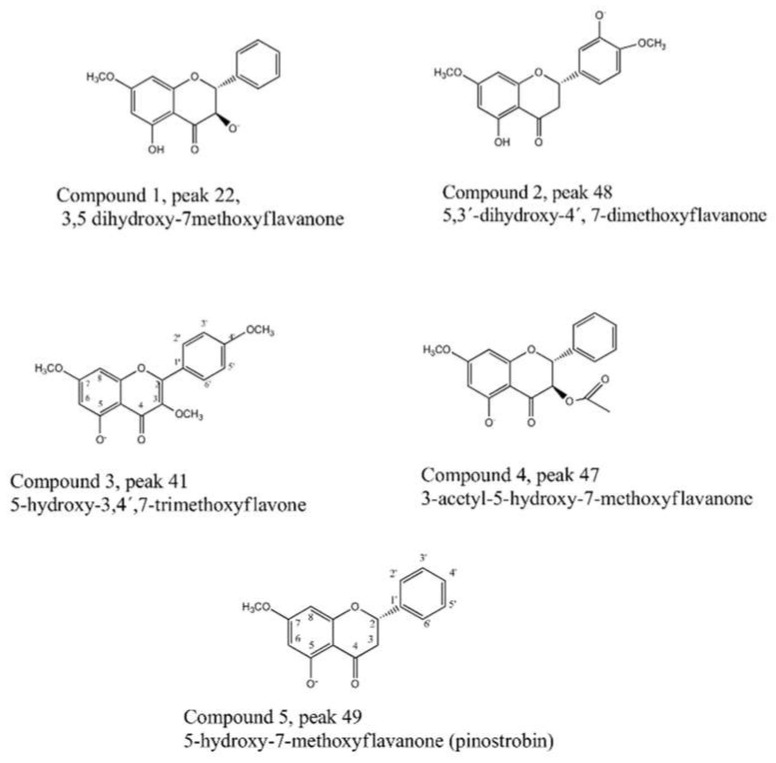
Structure of isolated flavonoids by high-speed counter-current chromatography (HSCCC).
