# Supplementary material for: Fast Isolation of Flavonoids from the Endemic Species Nolana ramosissima I.M. Johnst and Its Endothelium-Independent Relaxation Effect in Rat Aorta
Source: Molecules. 2020 Jan 24;25(3):520. doi: 10.3390/molecules25030520 (PMC7036828; doi:10.3390/molecules25030520)

Table S1. UHPLC-DAD-MS identification of metabolites in *N. ramossisima* polar extracts.

Peak #	RT (min.)	UV max (nm)	Tentative identification	Elemental composition [M-H]$^-$	Theoretical mass (*m/z*)	Measured mass (*m/z*)	Accuracy (δ ppm)	Type of compound	MS^n ions	Extract
1	1.75	-	2,4,5,6,7-Pentahydroxypentanoic acid	$C_7H_{13}O_7^-$	209.06668	209.06636	-1.53	Organic acid	191.05553<br>($C_7H_{11}O_6^-$)<br>Quinic acid	Me, Aq
2	1.95	230-346	Sculetin-5-hydroxyl-7-methoxyl-6-*O*-glucoside	$C_{18}H_{17}O_9^-$	377.08781	377.08542	-6.33	Coumarin		Me, Aq
3	3.61	230-346	Sculetin-6-*O*-glucoside (esculine)	$C_{15}H_{15}O_9^-$8.	339.07216	339.07178	-1.12	Coumarin glucoside	177.01874<br>($C_9H_5O_4^-$) sculetin<br>133.02873<br>($C_8H_5O_2^-$)	Aq
4	4.25	287	7-Methoxy-3-glucosyl flavanone	$C_{22}H_{23}O_{10}^-$	447.12967	447.12811	-3.48	Flavanone		Me
5	5.60	245	2,4-Dihydroxybenzoic acid	$C_7H_5O_4^-$	153.01933	153.01881	-3.39	Organic acid	108.02066<br>($C_7H_5O_2^-$,M -CO_2)	Me, Aq
6	6.28	230-346	5-Hydroxy-7-methoxyesculin	$C_{16}H_{17}O_{10}^-$	369.08272	369.08267	0.13	Coumarin glucoside	160.02374<br>($C_9H_5O_3^-$) umbelliferone	Me
7	7.82	287	Naringenin-4',7-dimethoxyl-3-*O*-rhamnoside	$C_{23}H_{25}O_{10}^-$	461.14532	461.14371	3.49	Flavanone glicoside		Me
8	8.25	234-303	Salicilic acid*	$C_7H_5O_3^-$	137.02442	137.02391	-3.72	Organic acid	108.02066<br>($C_7H_5O_2^-$, M-CO_2)	Me, Aq
9	9.21	287	Naringenin 4'-acetyl-7-methoxyl-3-*O*-rhamnoside	$C_{24}H_{25}O_{11}^-$	489.14024	489.13876	-3.02	Flavanone glicoside		Me, Aq
10	9.43	310	Caffeic acid*	$C_9H_7O_4^-$	179.03498	179.03497	-0.05	Organic acid	135.0439<br>($C_8H_7O_2^-$, M-CO_2)	Me
11	9.52	285	Eriodictyol 5-acetyl-3',4'-dimethoxyl-7- *O*-glucoside	$C_{25}H_{27}O_{11}^-$	503.15589	503.15442	2.92	Flavanone glicoside		Me
12	9.92	255-355	Quercetin-3-*O*-rutinoside*	$C_{27}H_{29}O_{16}^-$	609.14611	609.14606	-8.13	Flavonol glycoside	271.02475<br>$C_{14}H_7O_6^-$	Me, Aq
13	10.01	264-364	Kaempferol- 3-rutenoside	$C_{27}H_{29}O_{15}^-$	593.15112	593.15119	0.11	Flavonol glycoside	271.02475<br>$C_{14}H_7O_6^-$	Me, Aq
14	10.18	220	9,10-dihydroxy-6-oxodecanoic acid	$C_{10}H_{17}O_5^-$	217.10815	217.10805	-9.9	Fatty acid	109.02821<br>$C_6H_6O_2^-$	Me, Aq
15	10.36	287	Naringenin 3-hydroxyl-8-(3-methyl-2-butenyl) 7-*O*-glucoside	$C_{26}H_{29}O_{11}^-$	517.17154	517.17004	-2.9	Flavanone-glucoside		Me, Aq

16	10.88	227	Trihydroxy-octadecadienoic acid	$C_{18}H_{31}O_5^-$	343.21261	343.21320	1.71	Oxipilin		Me, Aq
17	10.96	287	Naringenin 3-Hydroxyl-4'-methoxyl-8-(3-methyl-2-butenyl) 7-glucoside	$C_{27}H_{31}O_{11}^-$	531.18719	531.18567	-2.86	Flavanone-glucoside		Me
18	11.48	230-346	Scopoletin*	$C_{10}H_7O_4^-$	191.03498	191.03490	-0.41	Coumarin		Me, Aq
19	11.96	255-355	Isorhamnetin- 3-rutinoside	$C_{28}H_{31}O_{16}^-$	623.16176	623.16132	-0.70	Flavonol glycoside	255.02950 $C_{14}H_7O_5^-$	Me, Aq
20	12.16	220	10-Hydroxy-6-oxodecanoic acid	$C_{10}H_{17}O_4^-$	201.11323	201.11302	-1.04	Fatty acid		Me, Aq
21	12.26	287	Naringenin 3,4'-Dimethoxyl-8-(3-methyl-2-butenyl) 7-*O*-glucoside	$C_{28}H_{33}O_{11}^-$	545.20284	545.20111	-10	Flavanone-glucoside		Me
22	12.32	287	3,5-Dihydroxy- 7-Methoxyflavanone	$C_{16}H_{13}O_5^-$	285.07685	285.07727	1.47	Flavanone	252.04295 $C_{15}H_8O_4^-$	Me, Aq
23	12.76	255-355	Quercetin-3-*O*-glucoside	$C_{27}H_{29}O_{16}^-$	463.08820	463.08847	0.58	Flavonol glicosidado	271.02460 $C_{14}H_7O_6^-$	Me, Aq
24	12.84	227	Trihydroxy-octadecadienoic acid	$C_{18}H_{31}O_5^-$	327.21770	327.21817	1.43	Oxilipin		Me, Aq
25	13.36	227	Dihydroxy-octadecadienoic acid-*O*-glucoside	$C_{24}H_{41}O_{10}^-$	489.27052	489.27121	1.41	Oxipilin		Me, Aq
26	13.50	220	Trihydroxy-octadecaenoic acid	$C_{18}H_{33}O_5^-$	329.23335	329.23386	1.09	Oxilipin		Me, Aq
27	13.86	220	9,10-Tetradecanoic acid	$C_{14}H_{27}O_4^-$	259.19148	259.19177	1.11	Oxilipin		Aq
28	14.15	220	9,10-Dihydroxy-hexadecanoic acid	$C_{16}H_{31}O_4^-$	287.22278	287.22321	1.49	Oxilipin		Me, Aq
29	14.36	227	Trihydroxy-octadecaenoic acid isomer	$C_{18}H_{33}O_5^-$	329,23335	329.23392	1.73	Oxilipin		Me, Aq
30	14.52	255-355	7,3'-Dimethoxyquercetin	$C_{17}H_{13}O_7^-$	329.06668	329.06680	0.36	Flavonol		Aq
31	14.85	-	Miristic acid-11-*O*-glucoside	$C_{20}H_{37}O_8^-$	405.24939	405.25012	2.49	Fatty acid		Me
32	15.75	287	5,7-Dihydroxyflavanone	$C_{15}H_{11}O_4^-$	255.06628	255.06656	1.09	Flavanone	240.04242 $C_{14}H_8O_4^-$	Me
33	15.82	220	Hydroxy-eicosaenoic acid glucoside	$C_{26}H_{47}O_9^-$	503.32256	503.32269	0.26	Oxilipin glucoside		Me
34	15.92	227	Hydroxy-hexadecaenoic acid-O-glucoside	$C_{22}H_{39}O_9^-$	447.26022	447.25996	0.58	Oxilipin glucoside		Me
35	16.26	227	Dihydroxy-octadecadienoic acid –O-glucoside	$C_{24}H_{41}O_{10}^-$	489.27052	489.27121	1.41	Oxipilin		Me, Aq
36	16.58	220	hydroxy-eicosaenoic acid glucoside	$C_{26}H_{45}O_9^-$	501.30691	501.30719	0.56	Oxilipin glucoside		Me, Aq

37	17.20	227	Dihydroxy-octadecadienoic acid	$C_{18}H_{31}O_4^-$	311.22278	311.22330	1.67	Oxipilin		Me, Aq
38	17.51	287	Dihydroxyflavanone isomer	$C_{15}H_{11}O_4^-$	255.06628	255.06612	-0.63	Flavanone	151.00296 $C_7H_3O_4^-$	Aq
39	18.00	269-337	7-Methoxyapigenin	$C_{16}H_{11}O_5^-$	283.06120	283.06158	0.23	Flavone	239.03494 $C_{14}H_7O_4^-$	Me, Aq
40	18.32	255-355	5,7-Dihydroxyflavone	$C_{15}H_9O_4^-$	253.05063	253.05040	0.91	Flavone	209.06020 $C_{14}H_9O_2^-$	Me, Aq
41	18.46	255-355	5-Hydroxy-3,4´,7-trimethoxyflavone	$C_{18}H_{15}O_6^-$	327.0874	327.0874	0	Flavone		Me, Aq
42	18.91	260-355	Kaempferol 7,4'-dimethyl ether	$C_{17}H_{13}O_6^-$	313.07176	313.07190	0.45	Flavonol	253.05074 $C_{15}H_9O_4^-$	Me
43	19.42	269- 337	7,4'-Dimethoxyapigenin	$C_{17}H_{13}O_5^-$	297.07685	297.07687	-0.07	Flavone	225.05515 $C_{14}H_9O_3^-$	Me, Aq
44	19.75	255-355	Quercetin*	$C_{15}H_9O_7^-$	301.03538	301.03546	-0.36	Flavonol	151.00307 ($C_7H_3O_4^-$)	Me, Aq
45	20.98	220	Hydroxy-docosanoic acid	$C_{22}H_{43}O_3^-$	355.32177	355.32230	-1.49	Oxipilin		Aq
46	19.95	285	3,5-Dihydroxy-7-methoxyflavanone	$C_{16}H_{13}O_5^-$	285.07685	285.07687	-0.07	Flavanone	252.04295 $C_{15}H_8O_4^-$	Me
47	20.32	287	3-Acetyl-5-hydroxy-7-methoxyflavanone	$C_{18}H_{15}O_6^-$	327.0874	327.0874	0	Flavanone		Me
48	20.52	287	5,3´-Dihydroxy-4´, 7-dimethoxyflavanone	$C_{17}H_{15}O_6^-$	315.08741	315.08743	0.12	Flavanone		Me, Aq
49	20.76	284	5-Hydroxy-7-methoxyflavanone (pinostrobin)	$C_{16}H_{13}O_4^-$	269.08193	269.08188	0.19	Flavanone	165.01819 $C_8H_5O_4^-$	Me, Aq
50	19.86	269- 337	Apigenin*	$C_{15}H_9O_5^-$	269.04555	269.04556	-0.04	Flavone		Me, Aq
51	21.32	220	Dihydroxy-octadecanoic acid	$C_{18}H_{35}O_4^-$	315.25408	315.25458	-1.40	Oxipilin		Me, Aq
52	22.01	232	Tetrahydroxy-eicosatrienoic acid	$C_{20}H_{33}O_6^-$	369.22826	369.22864	-1.03	Oxipilin		Me, Aq
53	22.54	227	Tetrahydroxy-eicosadienoic acid	$C_{20}H_{35}O_6^-$	371.24391	371.24423	-0.86	Oxipilin		Me
54	22.97	220	Trihydroxy-octadecenoic acid isomer	$C_{18}H_{33}O_5^-$	329.23335	329.23358	-0.70	Oxipilin	135.04440 $C_8H_7O_2^-$	Me, Aq
55	23.46	247	Tetrahydroxy-eicosatrienoic acid isomer	$C_{20}H_{33}O_6^-$	369.22826	369.22861	-0.95	Oxipilin		Me
56	23.47	287	Naringenin*	$C_{15}H_{11}O_5^-$	271.06120	271.06161	-1.51	Flavanone	211.03986 $C_{13}H_7O_3^-$	Me, Aq
57	25.50	243	Dihydroxy-octadecatrienoic acid	$C_{18}H_{29}O_4^-$	309.20713	309.20761	-1.55	Oxipilin	237.03978 $C_{11}H_9O_6^-$	Me, Aq
58	26.43	220	Dihydroxy-octadecenoic acid	$C_{18}H_{33}O_4^-$	313.23843	313.23889	1.46	Oxipilin	237.03978 $C_{11}H_9O_6^-$	Me

59	26.57	220	Dihydroxy-docosenoic acid	$C_{22}H_{41}O_4^-$	369.30103	369.30176	-.197	Oxipilin	Me, Aq
60	27.12	-	Hydroxymiristic acid	$C_{14}H_{27}O_3^-$	243.19657	243.19669	-0.49	Fatty acid	Me, Aq
61	27.57	-	Hydroxypalmitic acid	$C_{16}H_{31}O_3^-$	271.22787	271.22769	0.66	Organic acid	Me

*Identified by spiking experiments with authentic standards. Me: methanol, Aq: herbal tea.

Table S2. The k values of compounds **1**-**5** in the diphase solvent systems n-hexane/EtOAc/MeOH/H_2O and n-hexane/EtOAc/acetonitrile (measured by UHPLC).

**Solvent system**	**Compounds K Values**				
hexane/EtOAc/MeOH/H_2O	**1**	**2**	**3**	**4**	**5**
5:5:5:5	2.8	2.3	2.1	2.5	2.3
6:1:6:1	1.6	1.3	1.5	1.6	1.8
6:2:6:2	2.0	2.4	2.0	2.6	2.9
n-hexane: ethanol: water	**1**	**2**	**3**	**3**	**4**
7:4:1	0.78	1.42	1.7	1.53	1.9
6:5:1*	0.66	0.71	0.96	0.83	1.3
6:5:2	0.45	0.37	0.32	0.45	1.0
n-hexane: methanol	**1**	**2**	**3**	**3**	**4**
1:1	1.9	3.0	2.2	1.6	3.2

*: selected solvent system.

Figure S1. Quattro MK-7 HSCCC system, equipped with two HPLC pumps, UV detector and fraction collector.

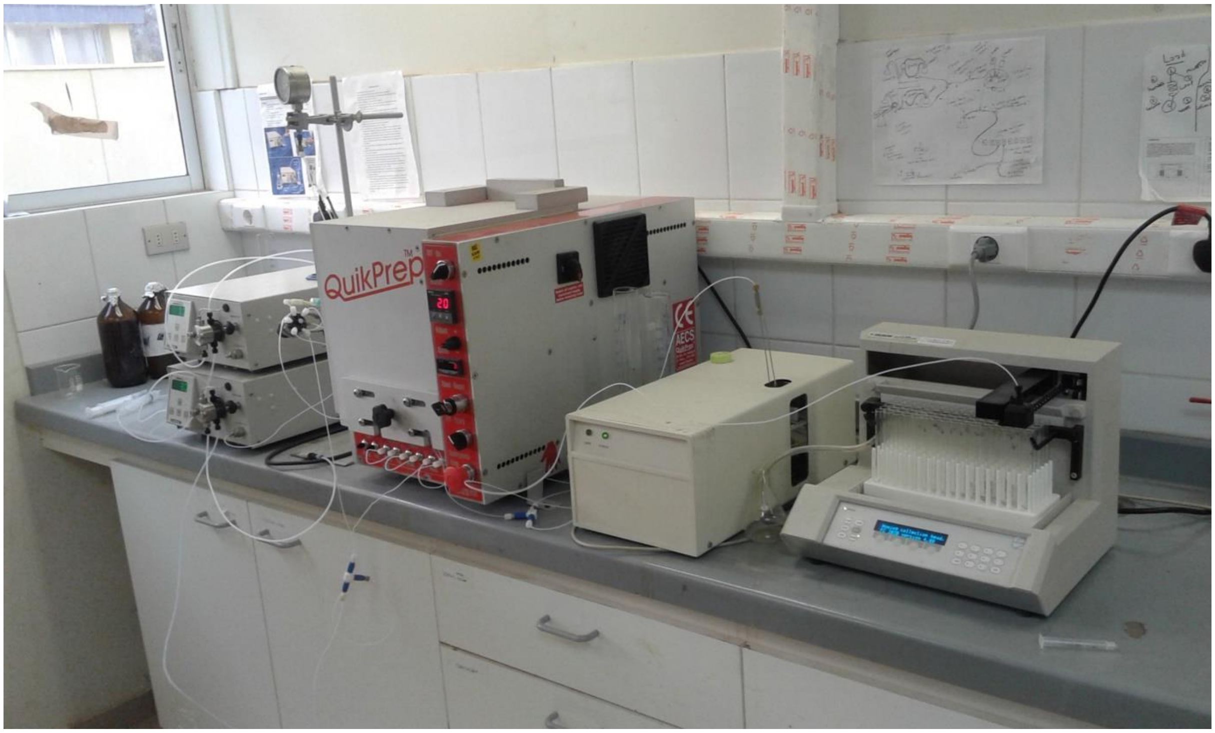

Figure S2. HSCCC chromatogram at 280 nm of *N. ramossisima* methanol extract.

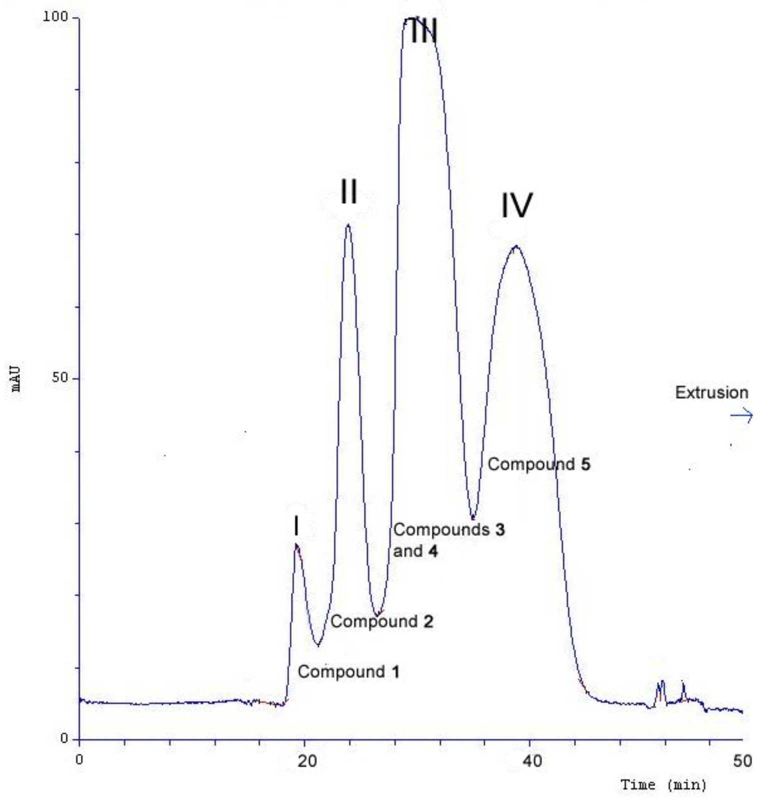

Figure S3. TLC plate (Silica gel F^{254}, developed with n-hexane:ethyl acetate 8:2 v/v and revealed with p-anysaldehyde-sulfuric acid and heating) of the HSCCC fractions from *N. ramosissima* methanol extract. (Only UV visible compounds **1**-**5** in elution mode).

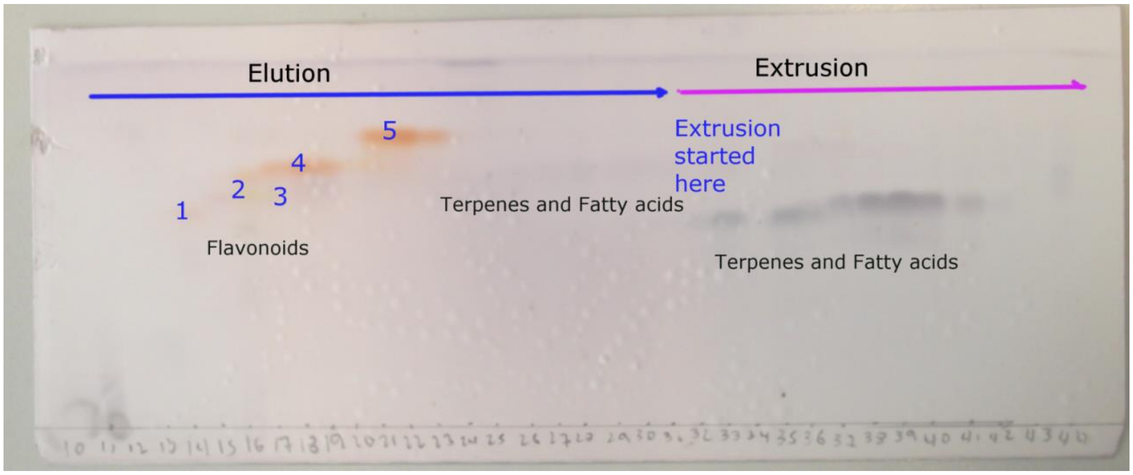

Figure S4. ^{1}H NMR (300 MHz) spectrum of Compound 1, peak 22, 3,5-dihydroxy-7methoxyflavanone.

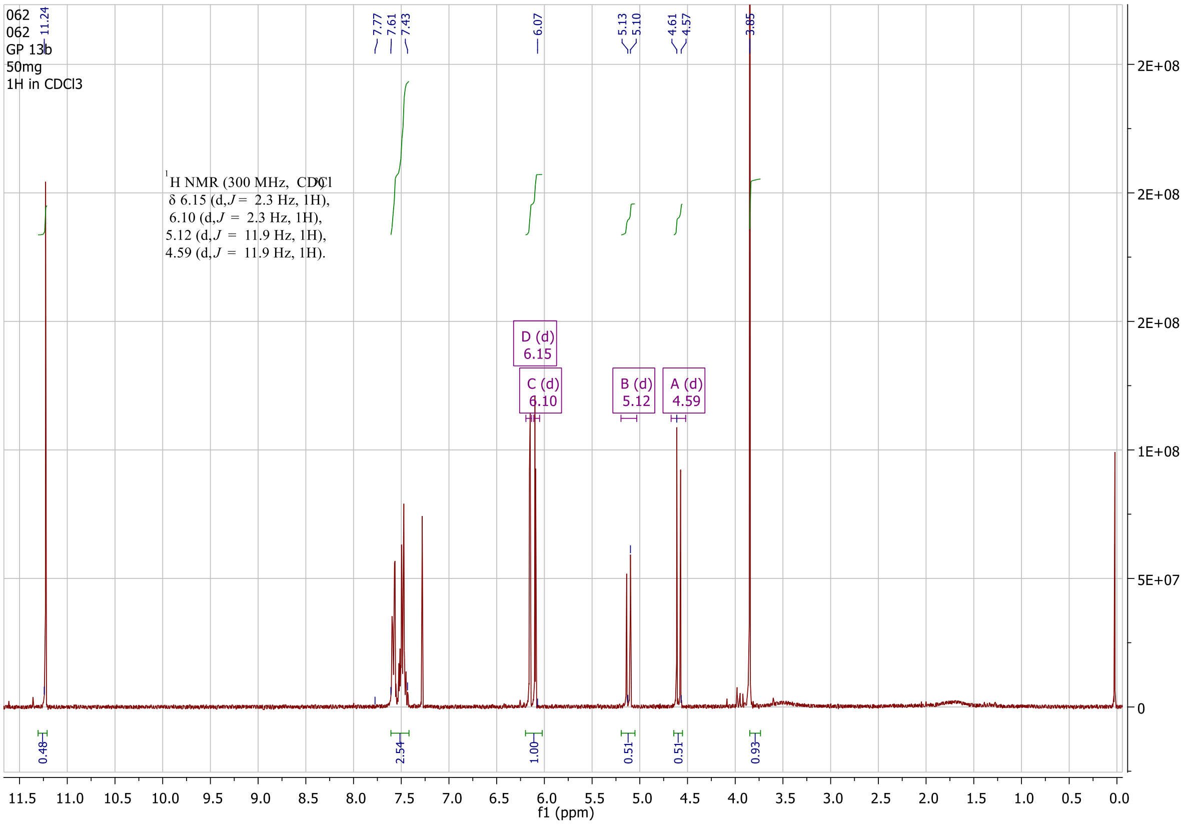

Figure S5. ^{1}H NMR (300 MHz) spectrum of Compound 1, peak 22, 3,5-dihydroxy-7methoxyflavanone. (Ampliation).

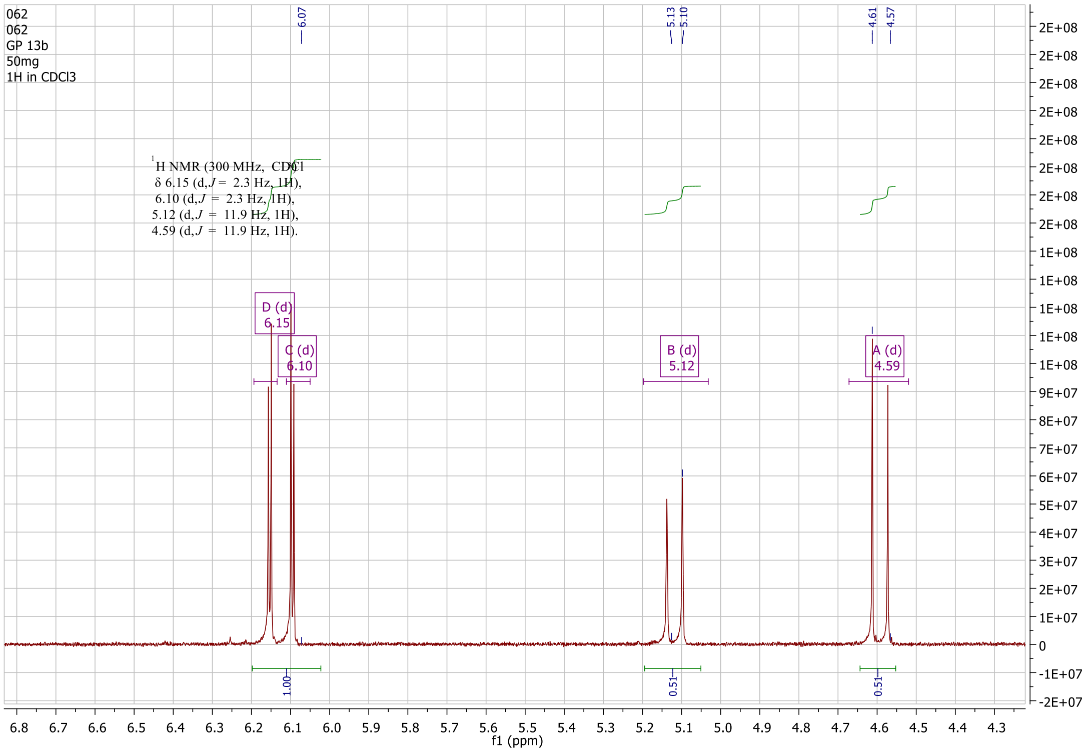

Figure S6. ^{1}H-^{1}H-COSY NMR spectrum (300 MHz) of Compound 1, peak 22, 3,5-dihydroxy-7methoxyflavanone.

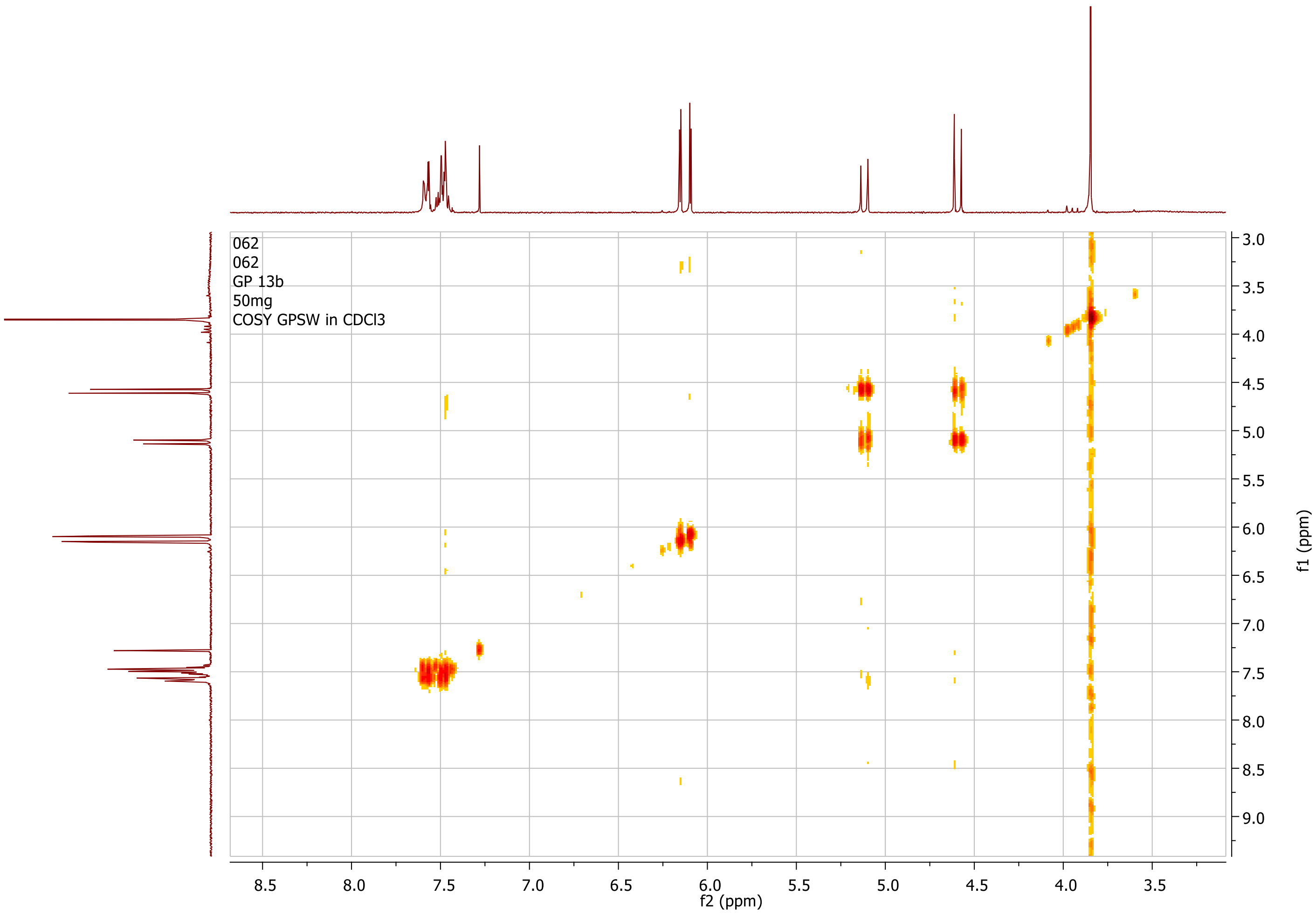

Figure S7. ^{13}C NMR spectrum (150.09 MHz) of Compound 1, peak 22, 3,5-dihydroxy-7methoxyflavanone.

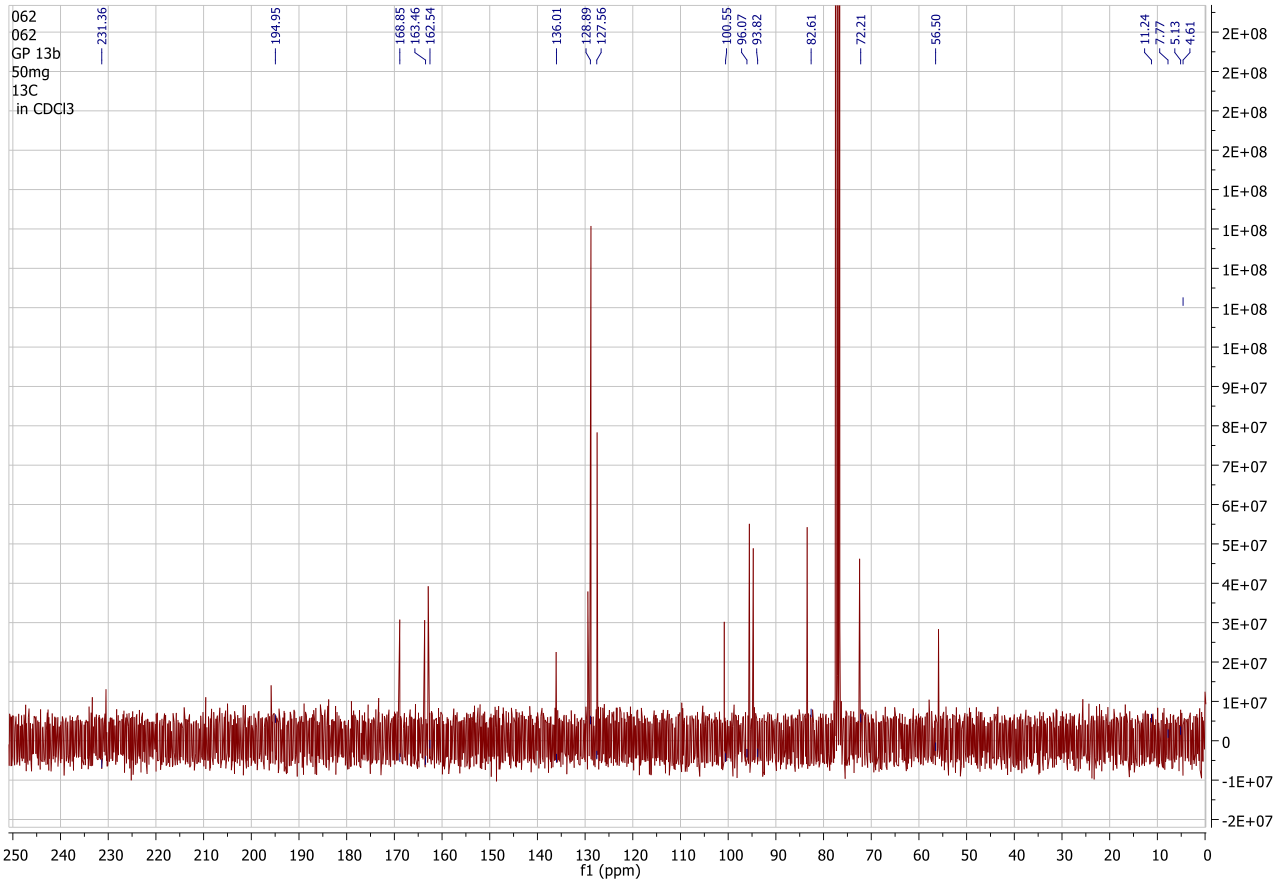

Figure S8. DEPT 135 NMR spectrum (150.09 MHz) of Compound 1, peak 22, 3,5-dihydroxy-7methoxyflavanone.

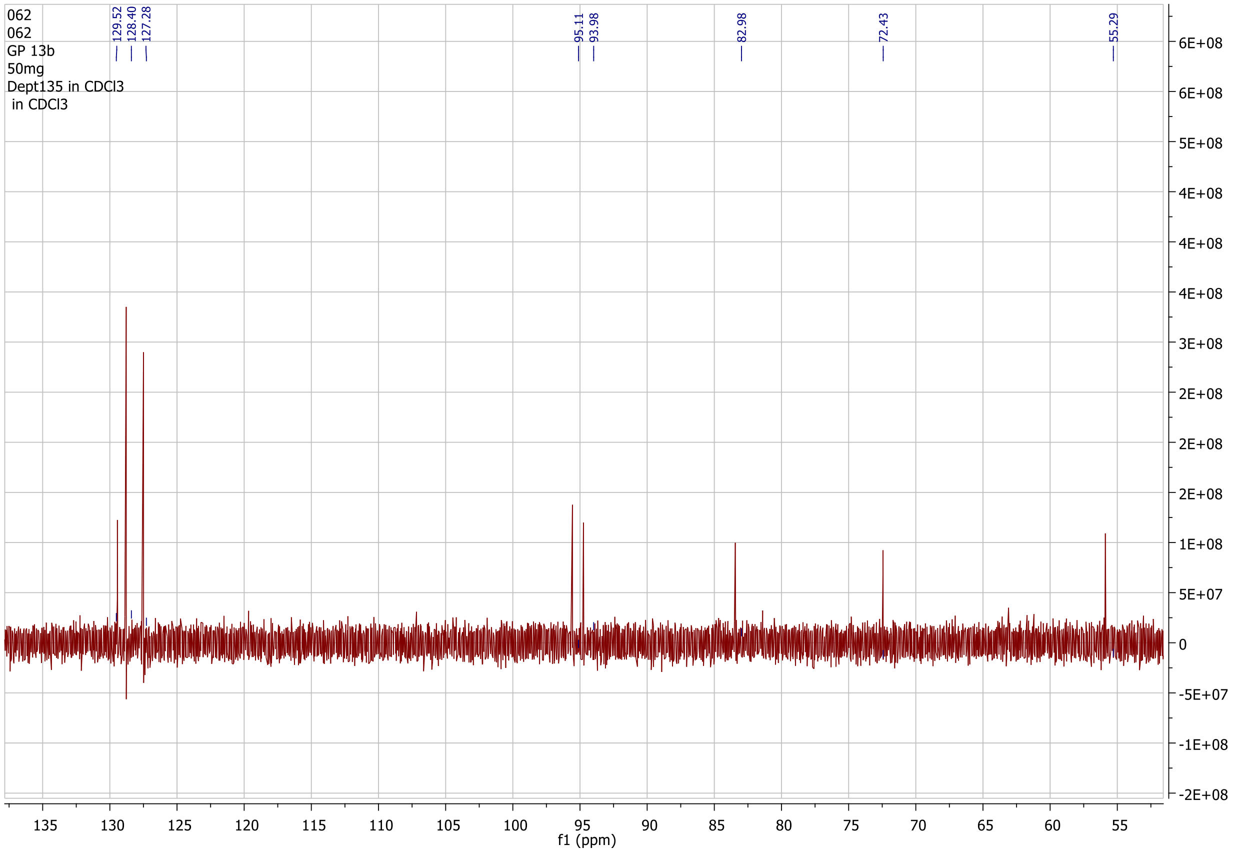

Figure S9. HMBC NMR spectrum (300 and 150.09 MHz) of Compound 1, peak 22, 3,5 dihydroxy-7methoxyflavanone.

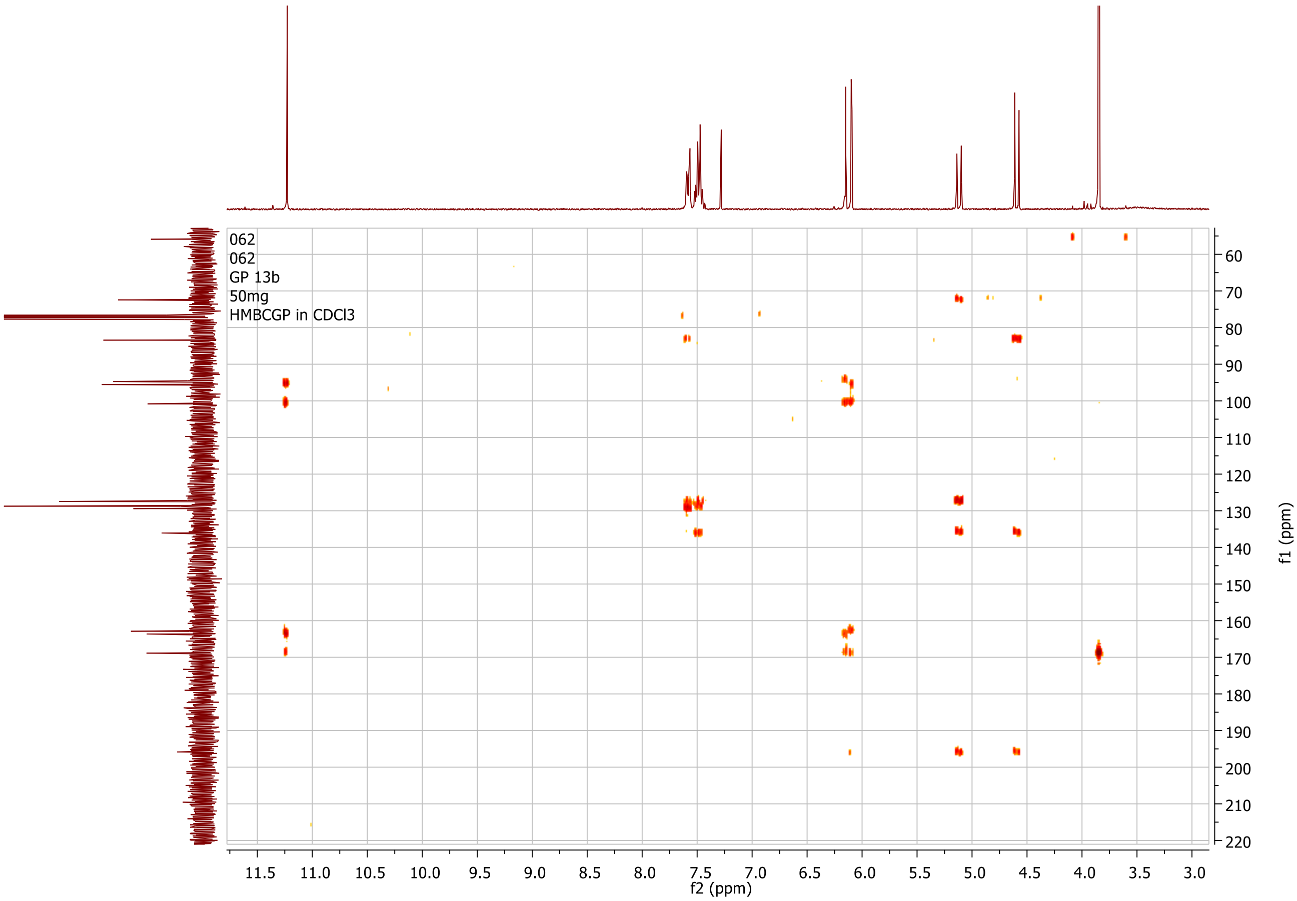

Figure S10. ^{1}H NMR (300 MHz) spectrum of Compound 2, peak 48, 5,3´-dihydroxy-4´, 7-dimethoxyflavanone.

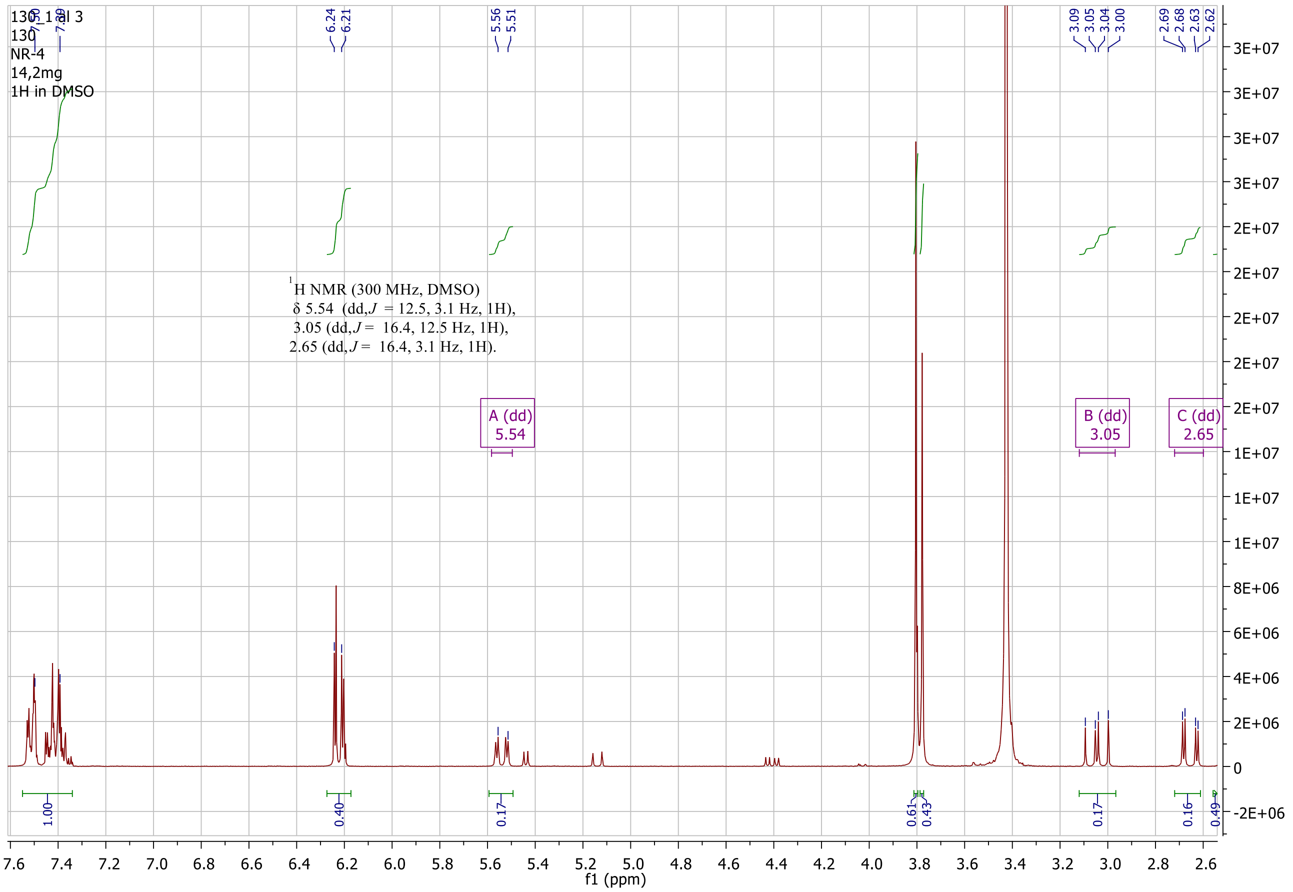

Figure S11. ^{13}C NMR spectrum (150.09 MHz) of Compound 2, peak 48, 5,3′-dihydroxy-4′, 7-dimethoxyflavanone.

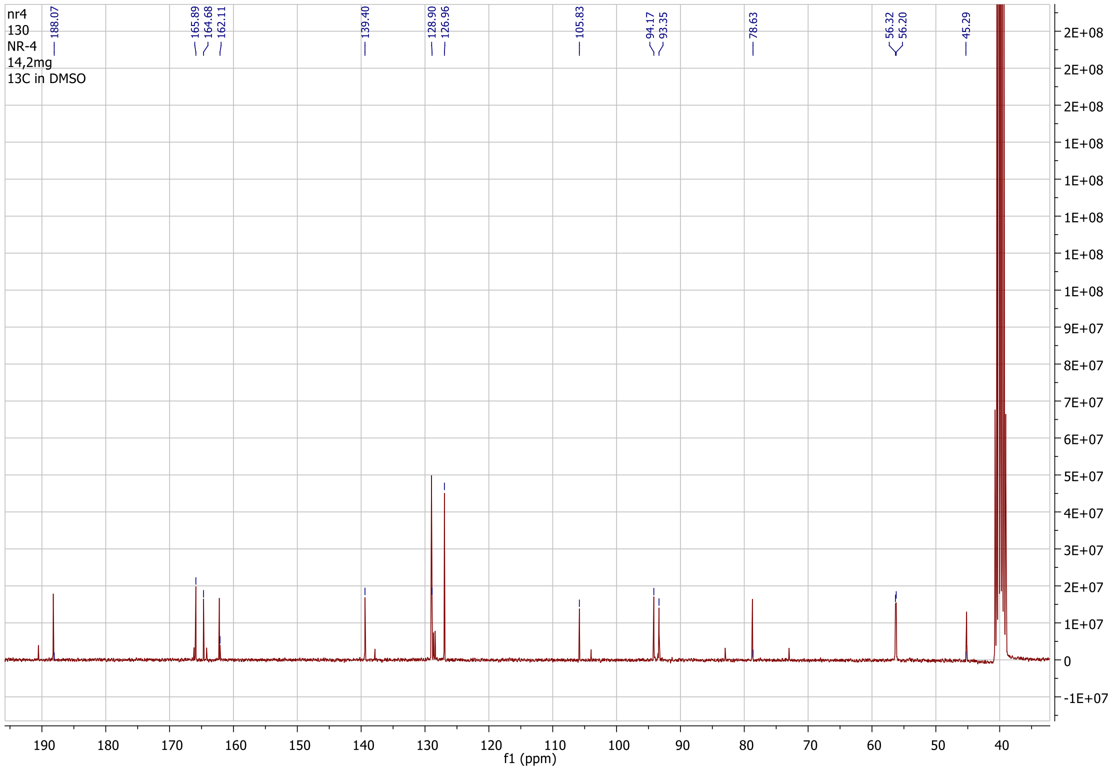

Figure S12. DEPT 135 NMR spectrum (150.09 MHz) of Compound 2, peak 48, 5,3′-dihydroxy-4′, 7-dimethoxyflavanone.

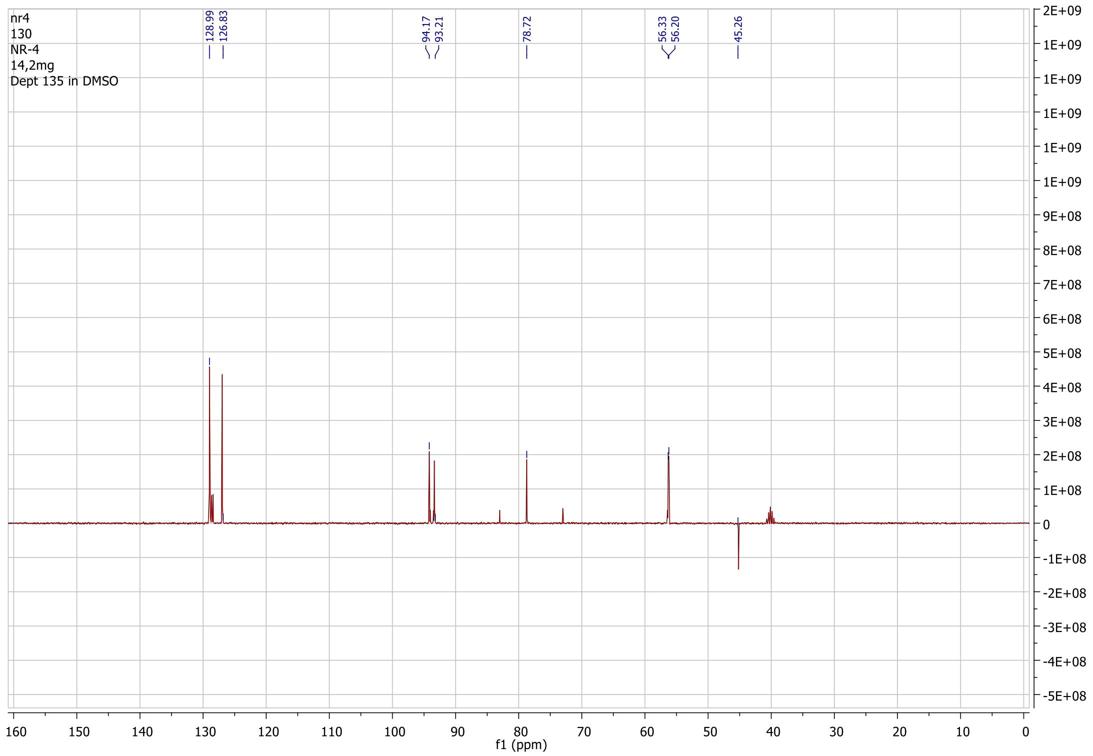

Figure S13. ^1H-^1H-COSY NMR spectrum (300 MHz) of Compound 2, peak 48, 5,3′-dihydroxy-4′, 7-dimethoxyflavanone.

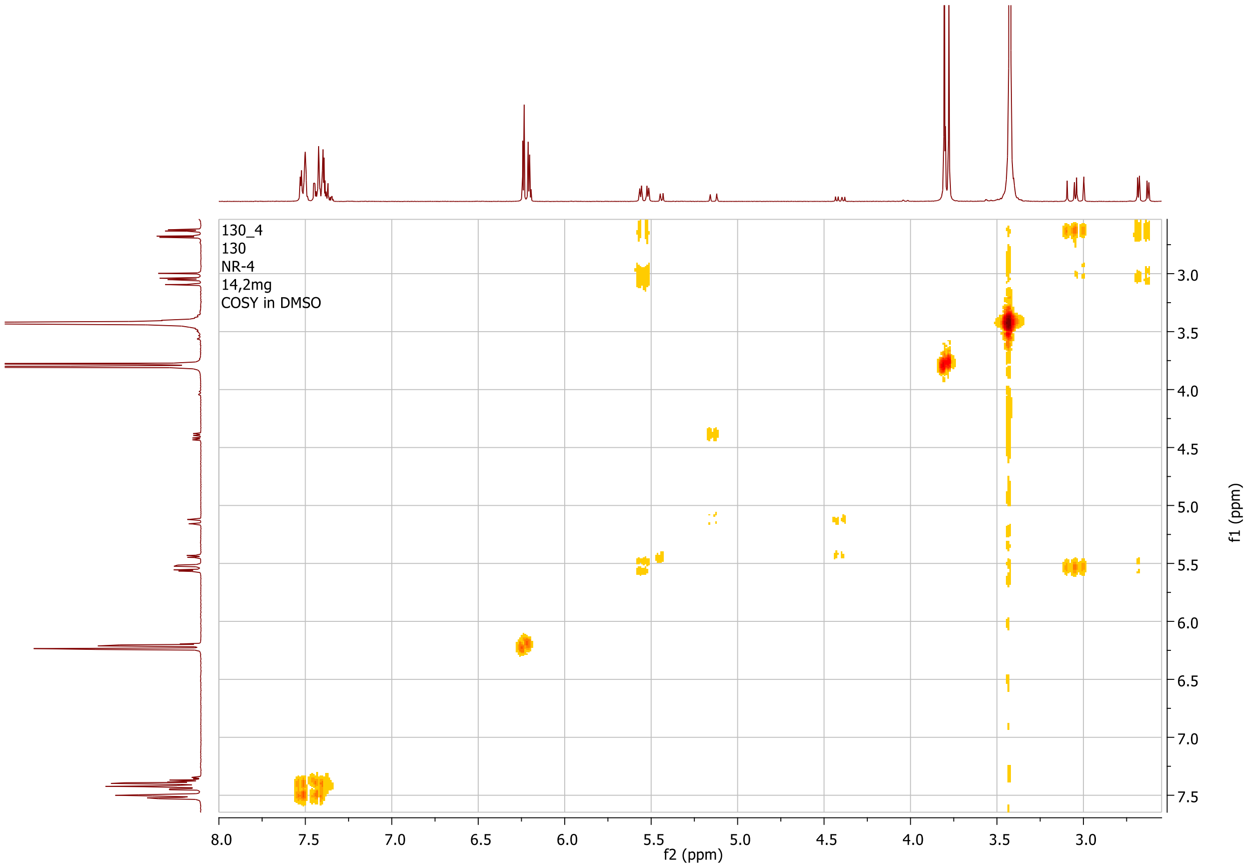

Figure S14. Phase edited HSQC NMR spectrum (300 and 150.09 MHz) of Compound 2, peak 48, 5,3′-dihydroxy-4′, 7-dimethoxyflavanone.

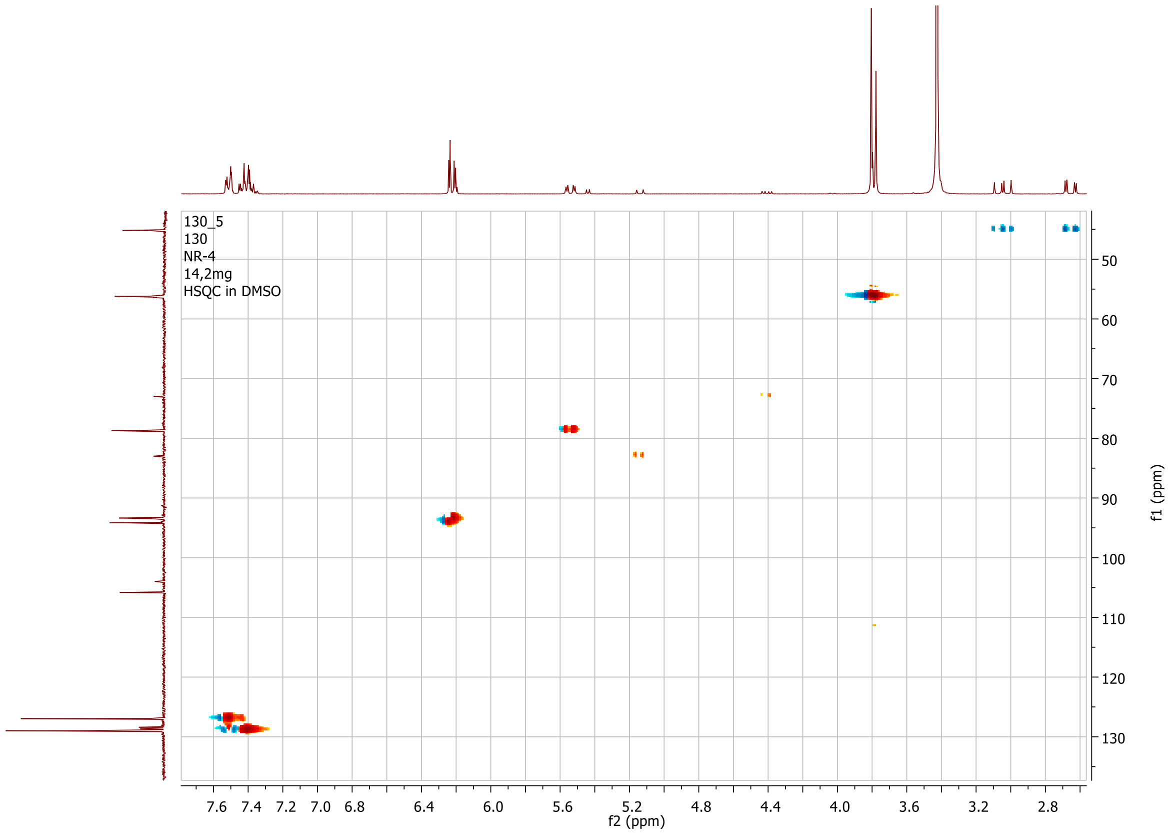

Figure S15. HMBC NMR spectrum (300 and 150.09 MHz) of Compound 2, peak 48, 5,3′-dihydroxy-4′, 7-dimethoxyflavanone.

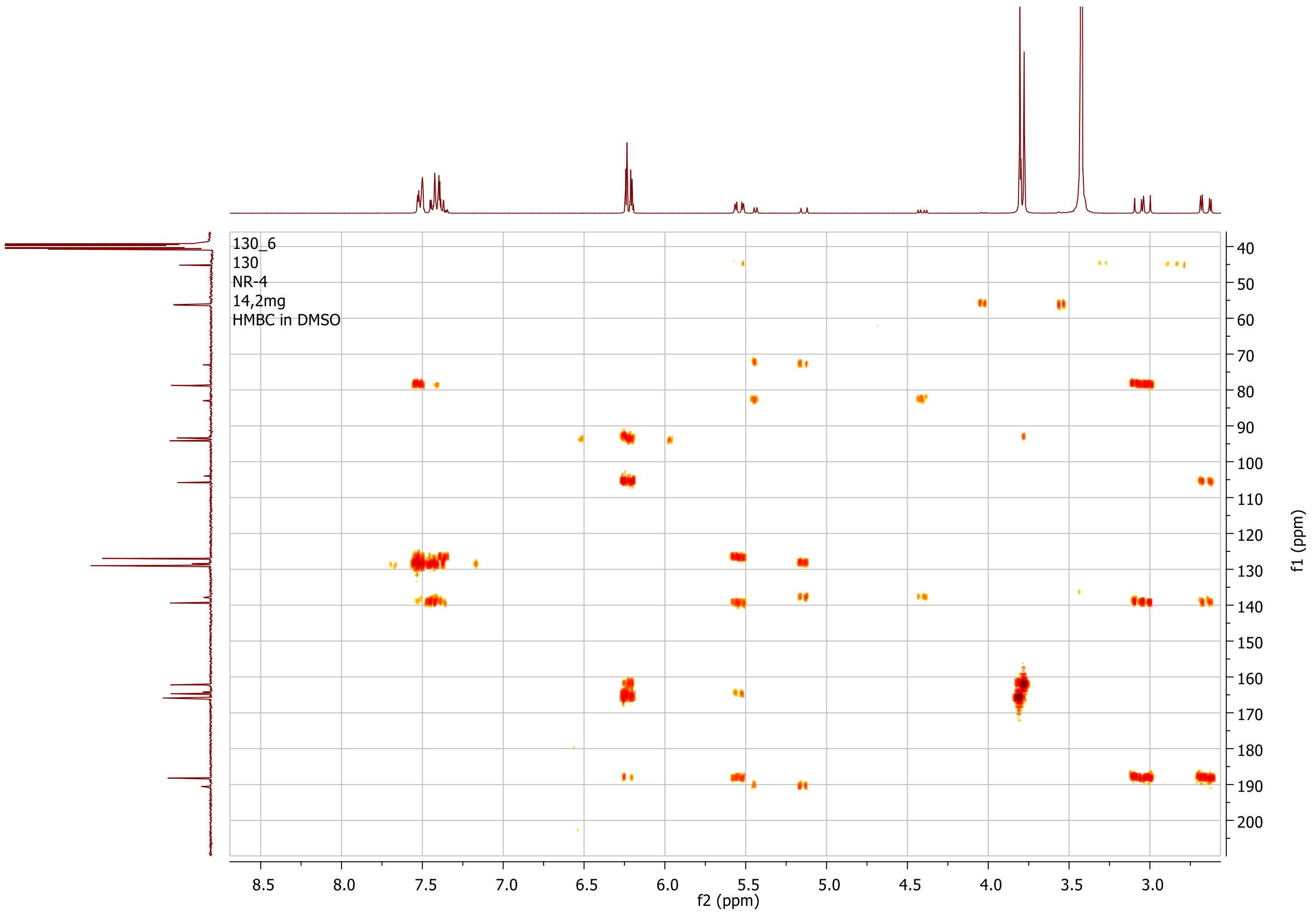

Figure S16. ^{1}H NMR (300 MHz) spectrum of Compound 3, peak 41, 5-hydroxy-3,4′,7-trimethoxyflavone .

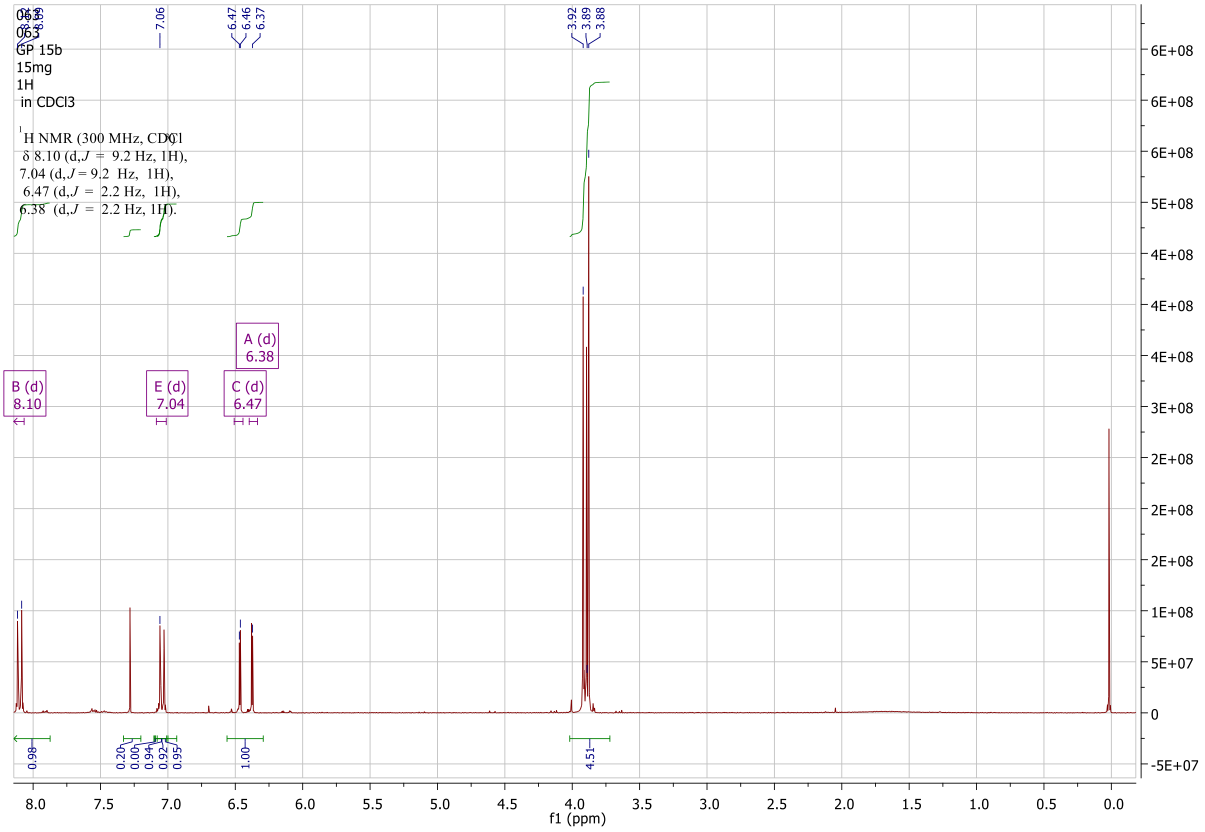

Figure S17. ^{1}H NMR (300 MHz) spectrum of Compound 3, peak 41, 5-hydroxy-3,4′,7-trimethoxyflavone (Ampliation).

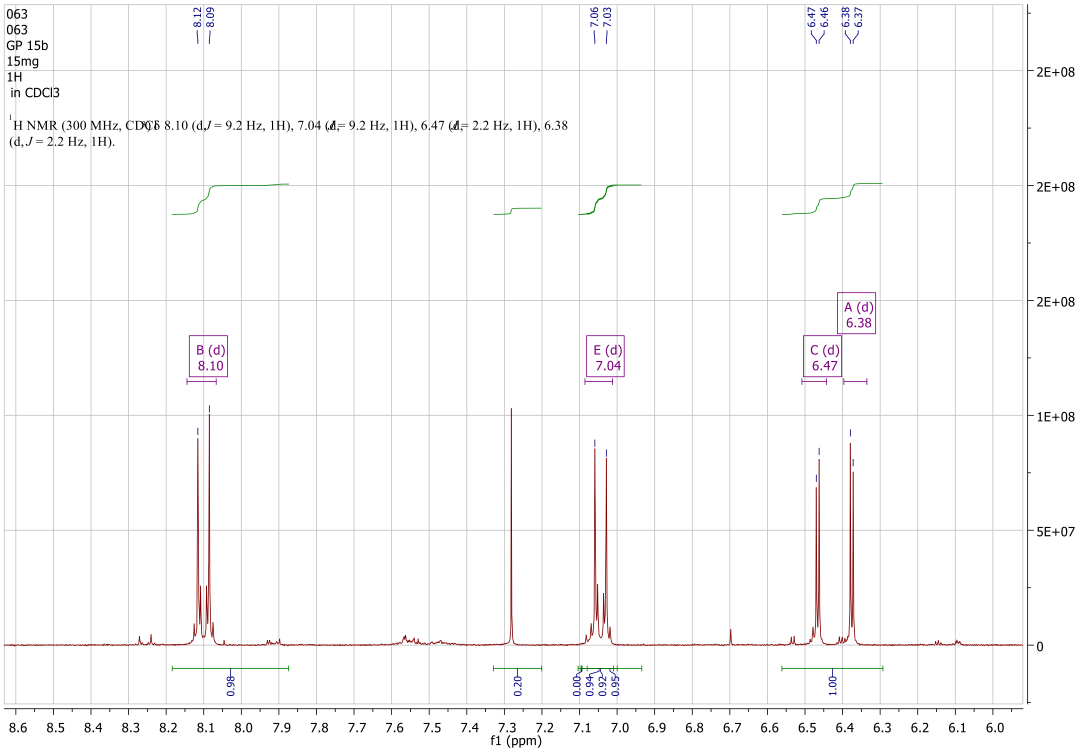

Figure S18. ^{13}C NMR spectrum (150.09 MHz) of Compound 3, peak 41, 5-hydroxy-3,4′,7-trimethoxyflavone.

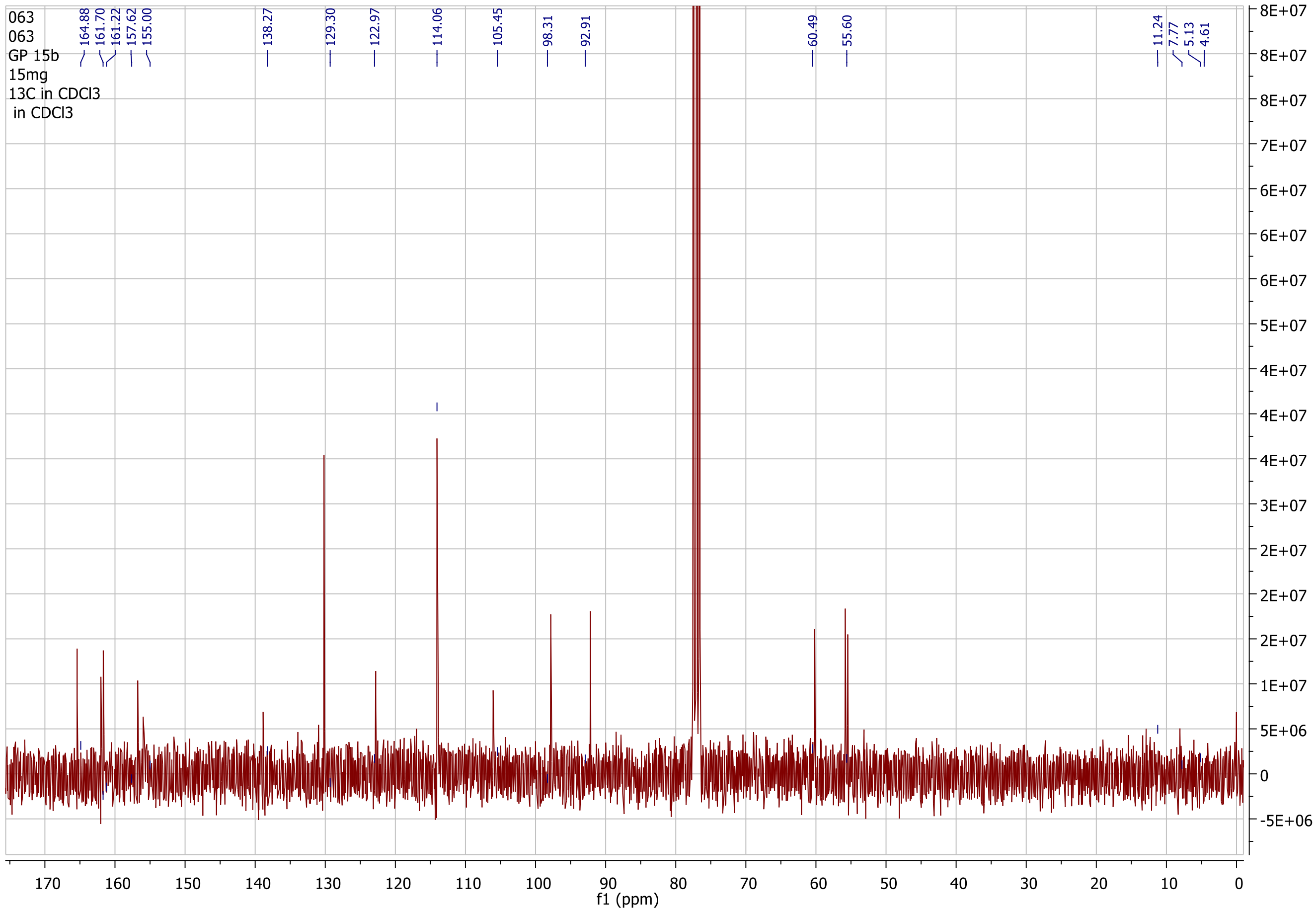

Figure S19. DEPT 135 NMR spectrum (150.09 MHz) of Compound 3, peak 41, 5-hydroxy-3,4′,7-trimethoxyflavone.

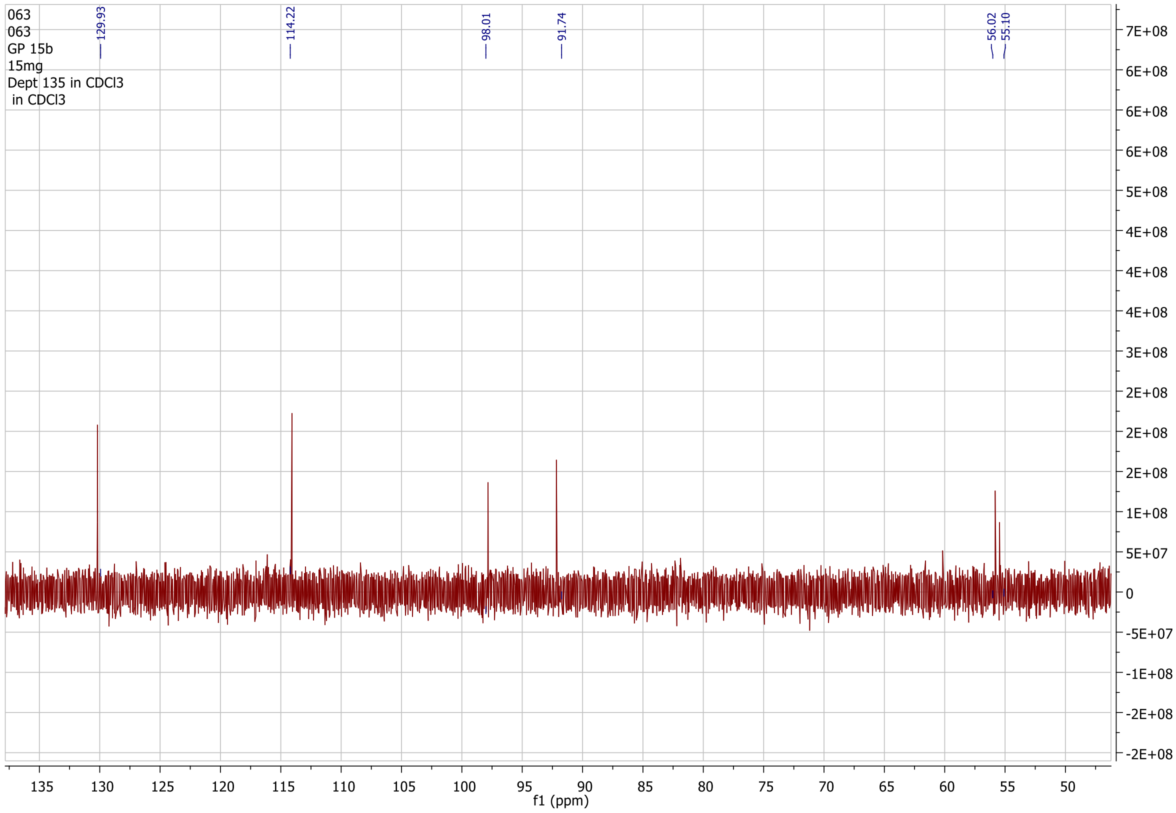

Figure S20. HSQC NMR spectrum (300 and 150.09 MHz) of Compound 3, peak 41, 5-hydroxy-3,4′,7-trimethoxyflavone.

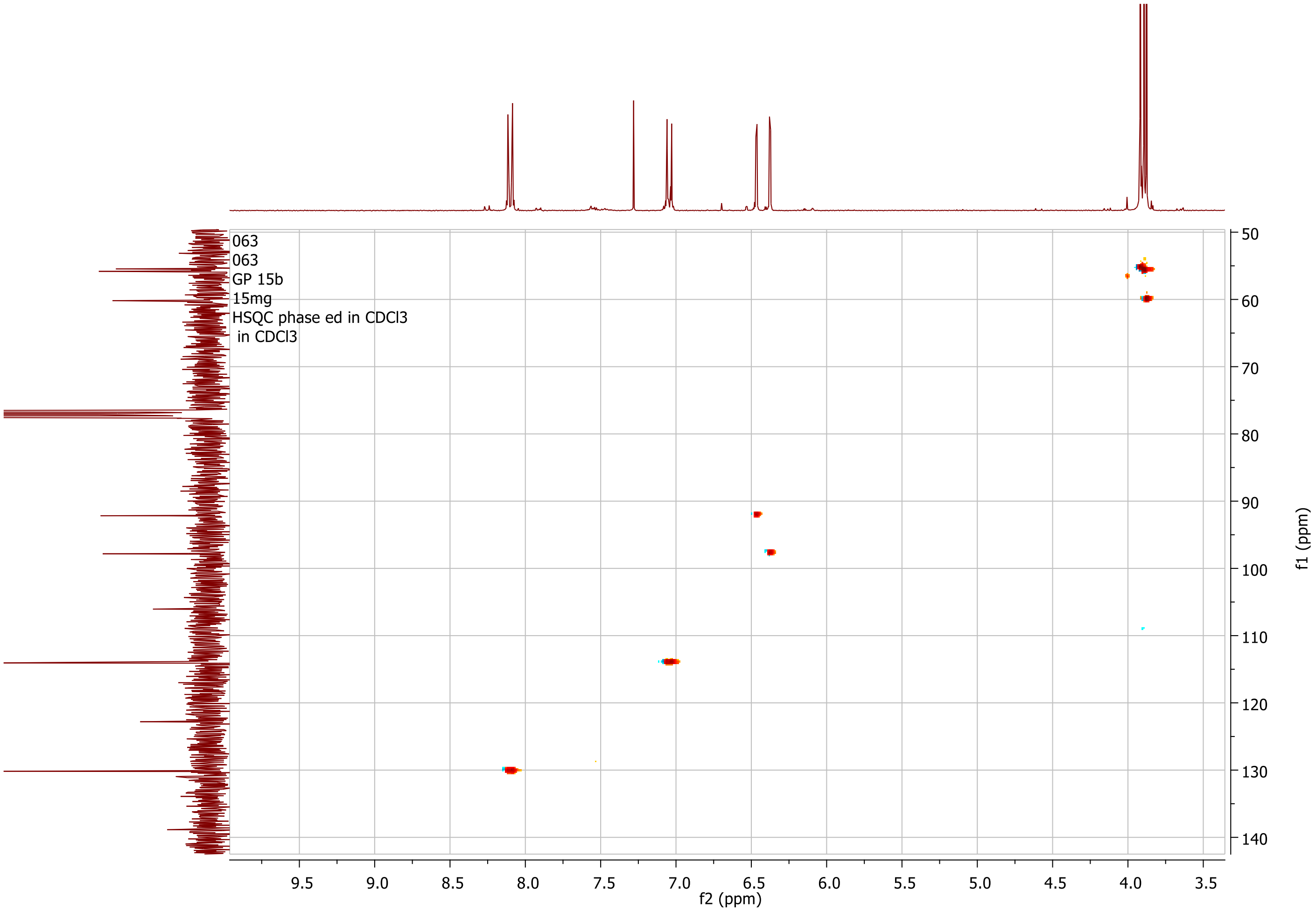

Figure S21. ^{1}H NMR (300 MHz) spectrum of compound Compound 4, peak 47, 3-acetyl-5-hydroxy-7-methoxyflavanone.

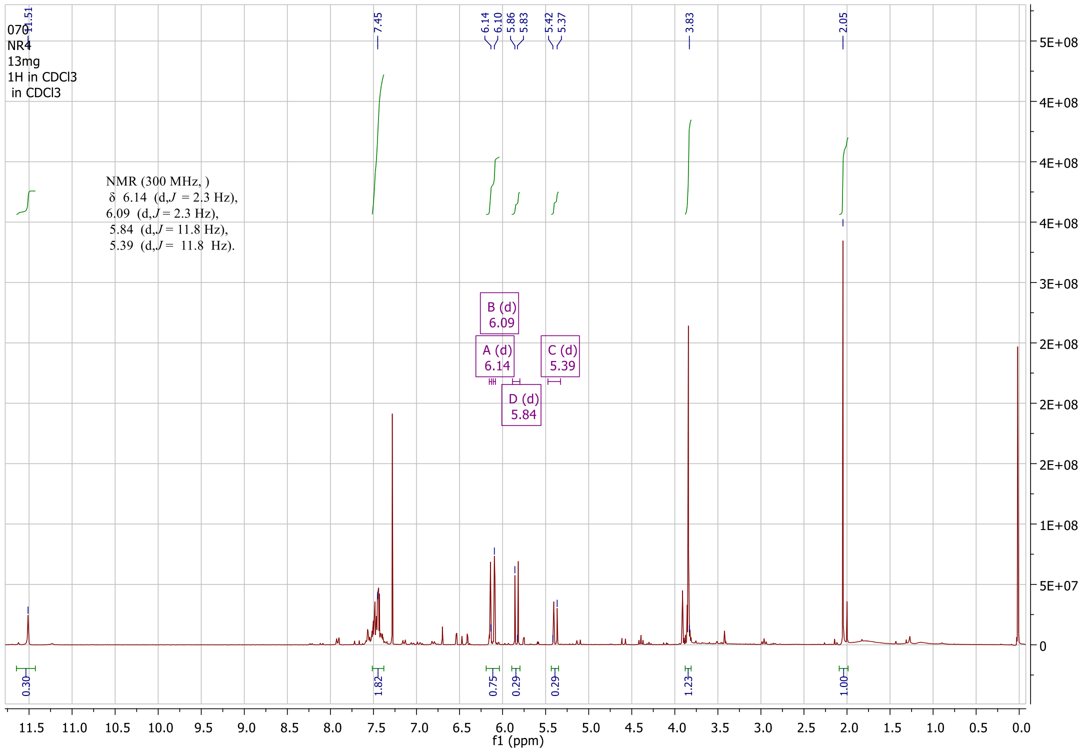

Figure S22. ^{13}C NMR spectrum (150.09 MHz) of Compound 4, peak 47, 3-acetyl-5-hydroxy-7-methoxyflavanone.

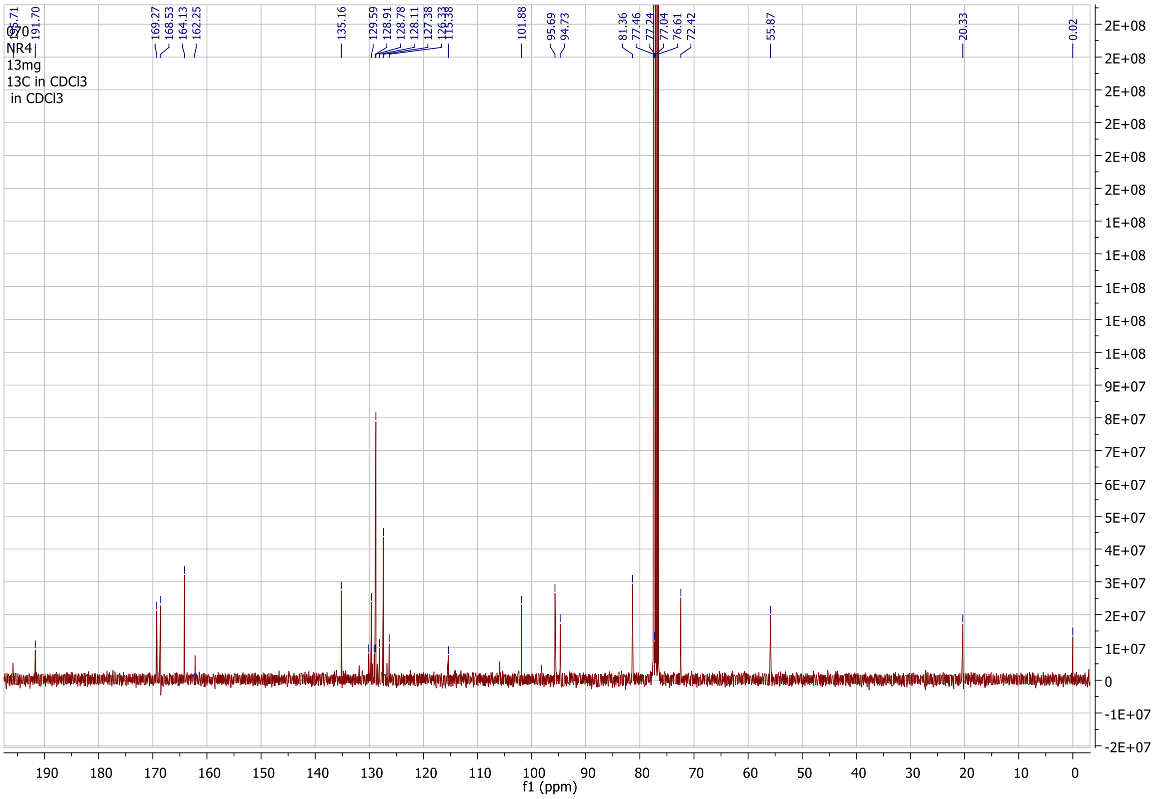

Figure S23. DEPT 135 NMR spectrum (150.09 MHz) of Compound 4, peak 47, 3-acetyl-5-hydroxy-7-methoxyflavanone.

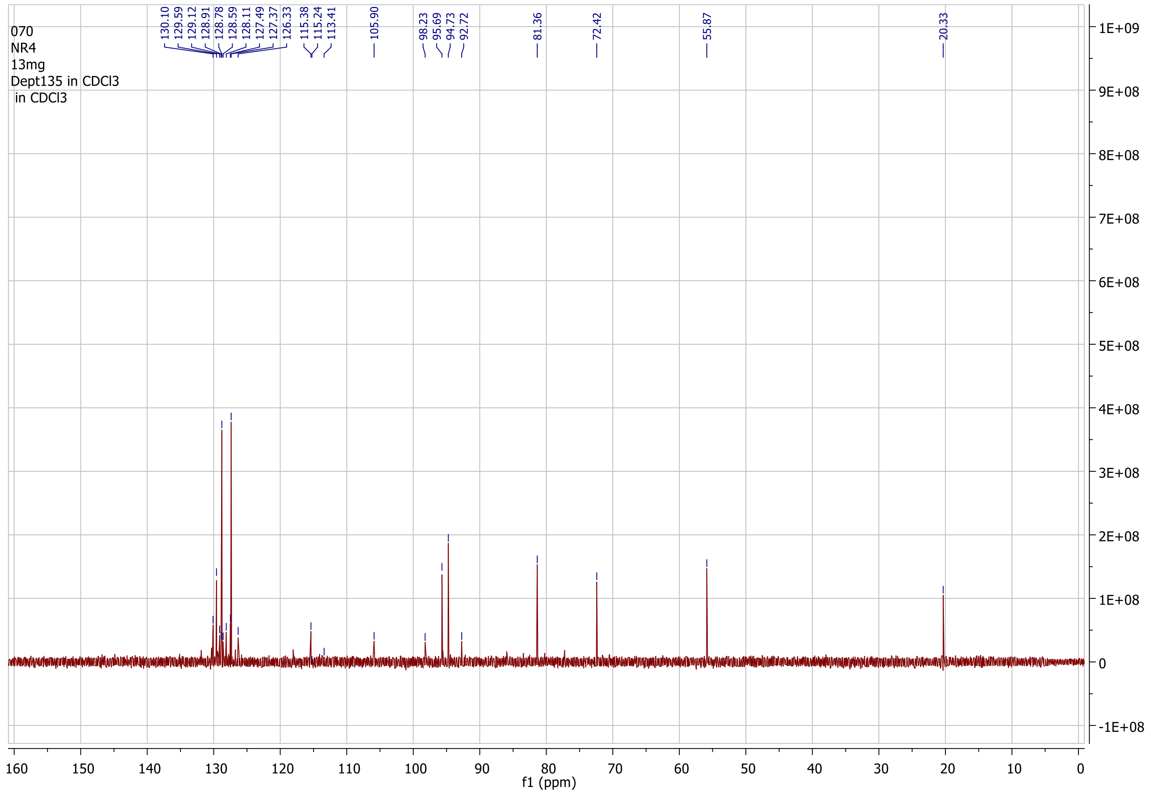

Figure S24. ^{1}H-^{1}H-COSY NMR spectrum (300 MHz) of Compound 4, peak 47, 3-acetyl-5-hydroxy-7-methoxyflavanone.

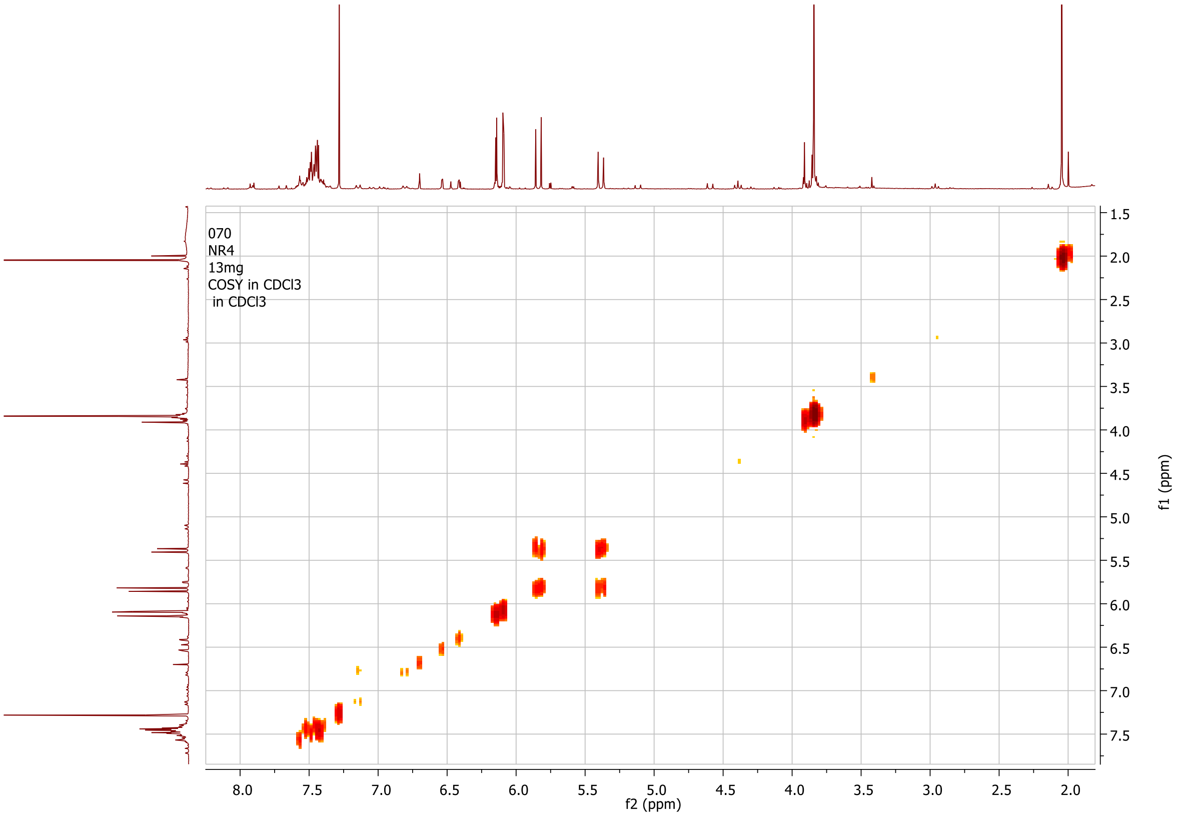

Figure S25. Phase edited HMBC NMR spectrum (300 and 150.09 MHz) of Compound 4, peak 47, 3-acetyl-5-hydroxy-7-methoxyflavanone.

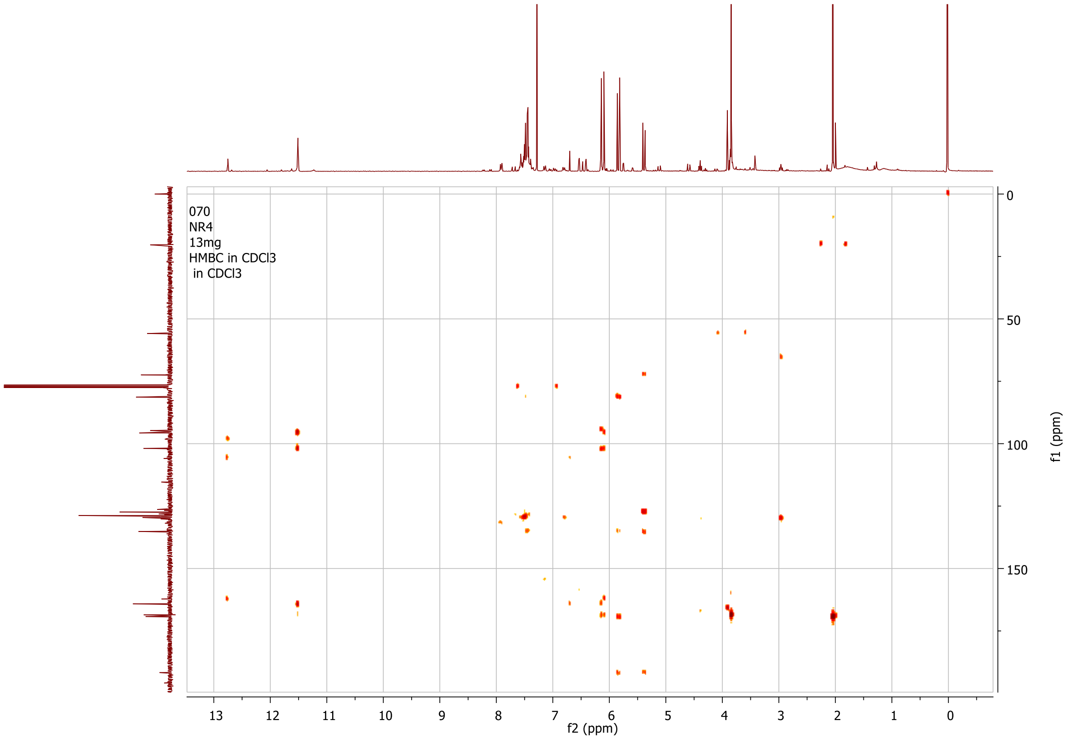

Figure S26. Phase edited HMBC NMR spectrum (300 and 150.09 MHz) of Compound 4, peak 47, 3-acetyl-5-hydroxy-7-methoxyflavanone.

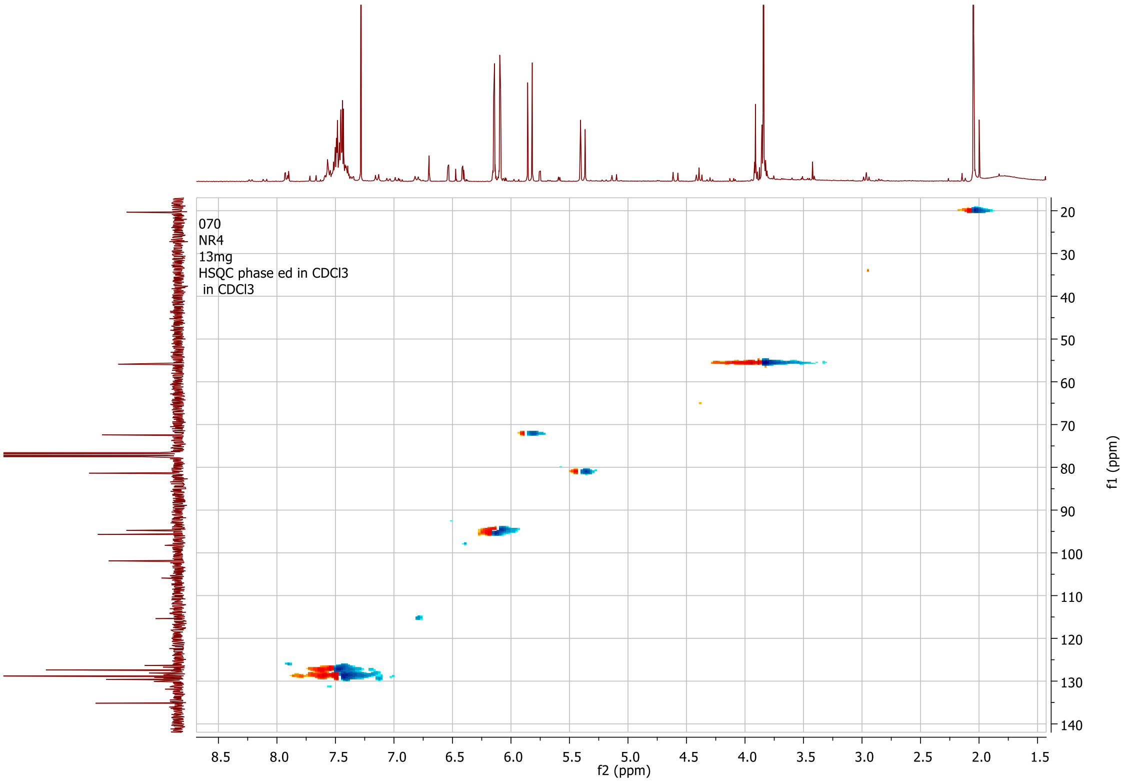

Figure S27. ^{1}H NMR (300 MHz) spectrum of Compound 5, peak 49, 5-hydroxy-7-methoxyflavanone (pinostrobin).

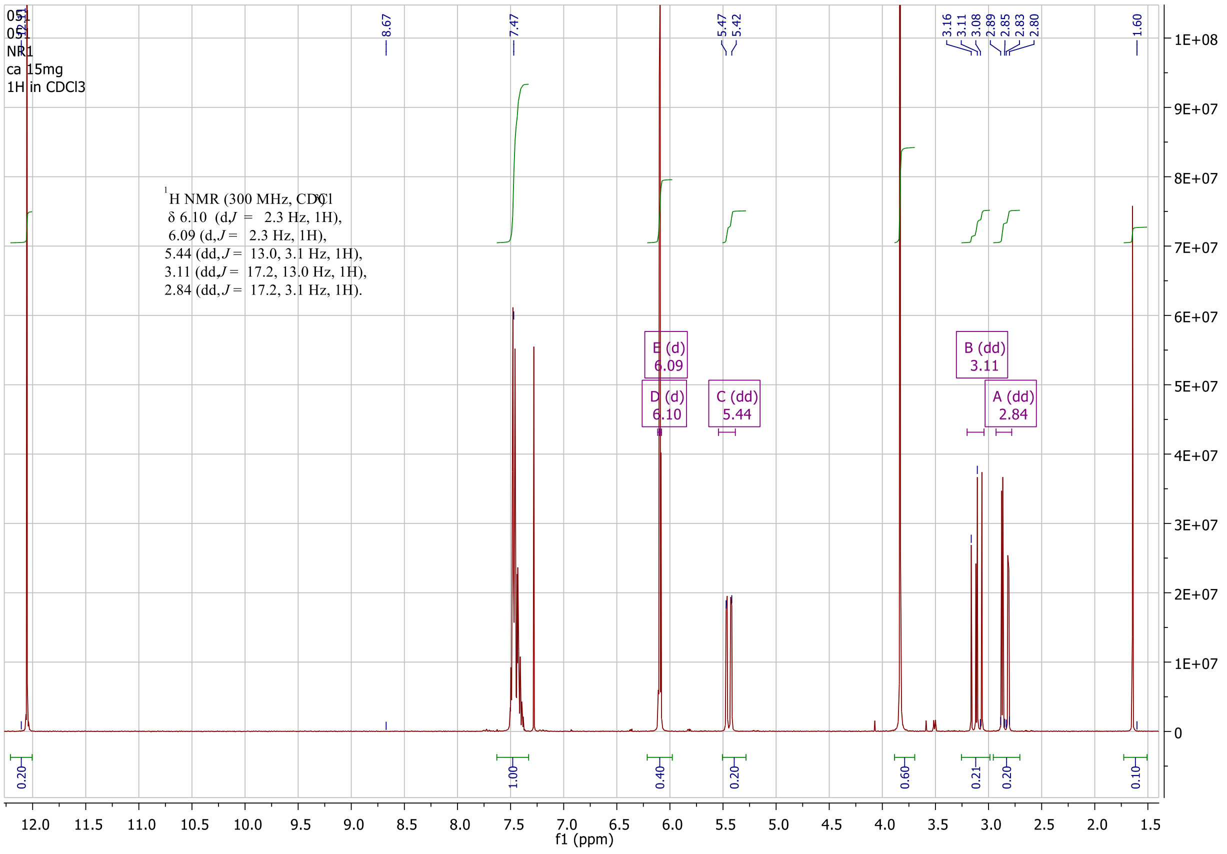

Figure S28. ^{1}H NMR (300 MHz) spectrum of Compound 5, peak 49, 5-hydroxy-7-methoxyflavanone (pinostrobin).(Ampliation).

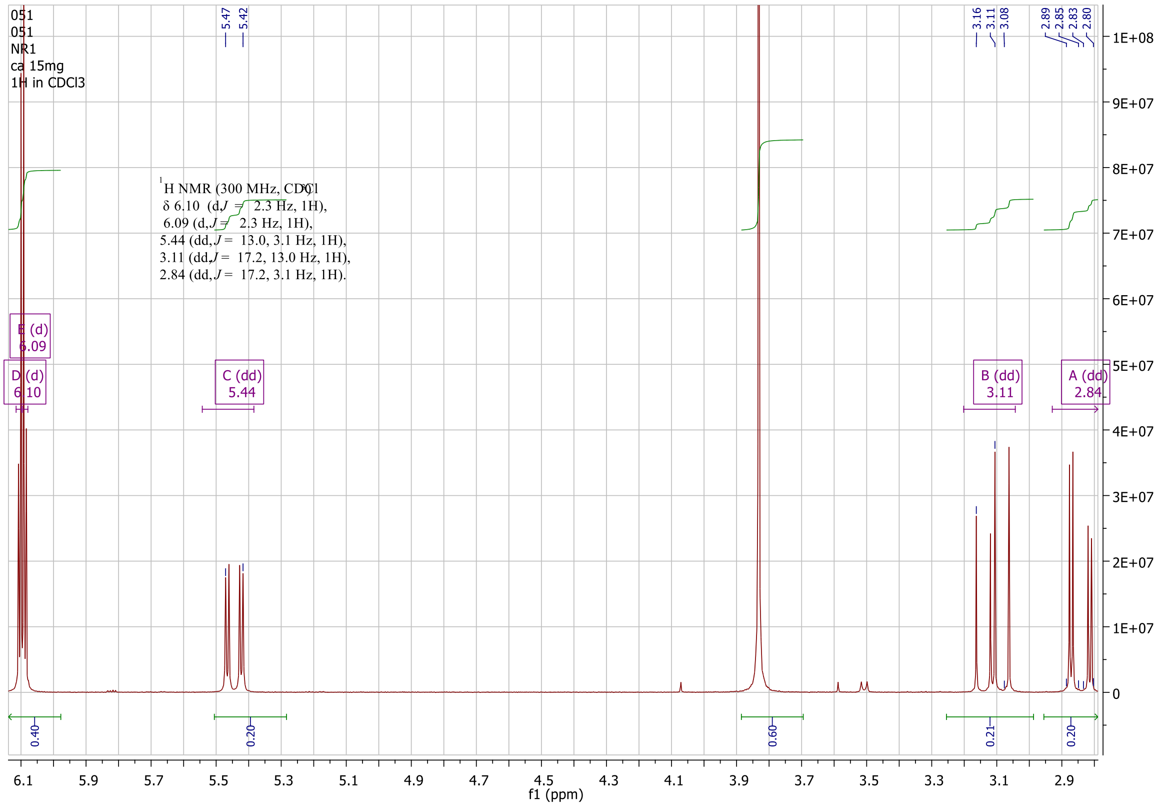

Figure S29. C NMR spectrum (150.09 MHz) of Compound 5, peak 49, 5-hydroxy-7-methoxyflavanone (pinostrobin).

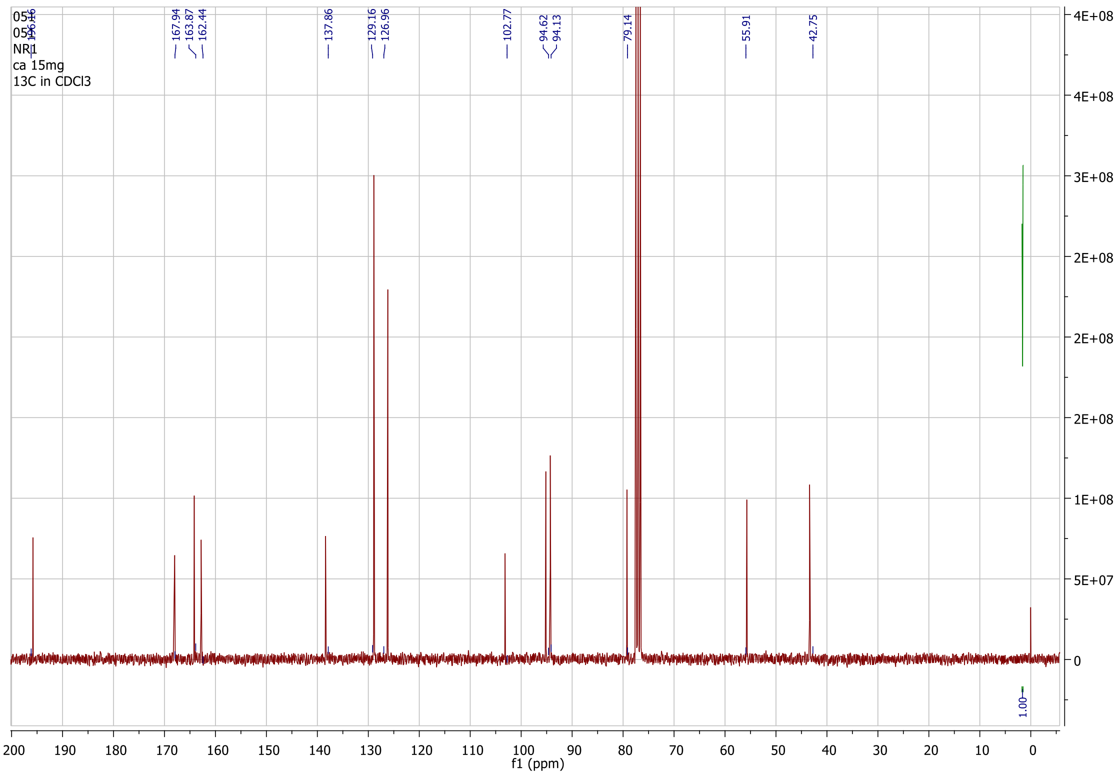

Figure 30. DEPT 135 NMR spectrum (150.09 MHz) of Compound 5, peak 49, 5-hydroxy-7-methoxyflavanone (pinostrobin).

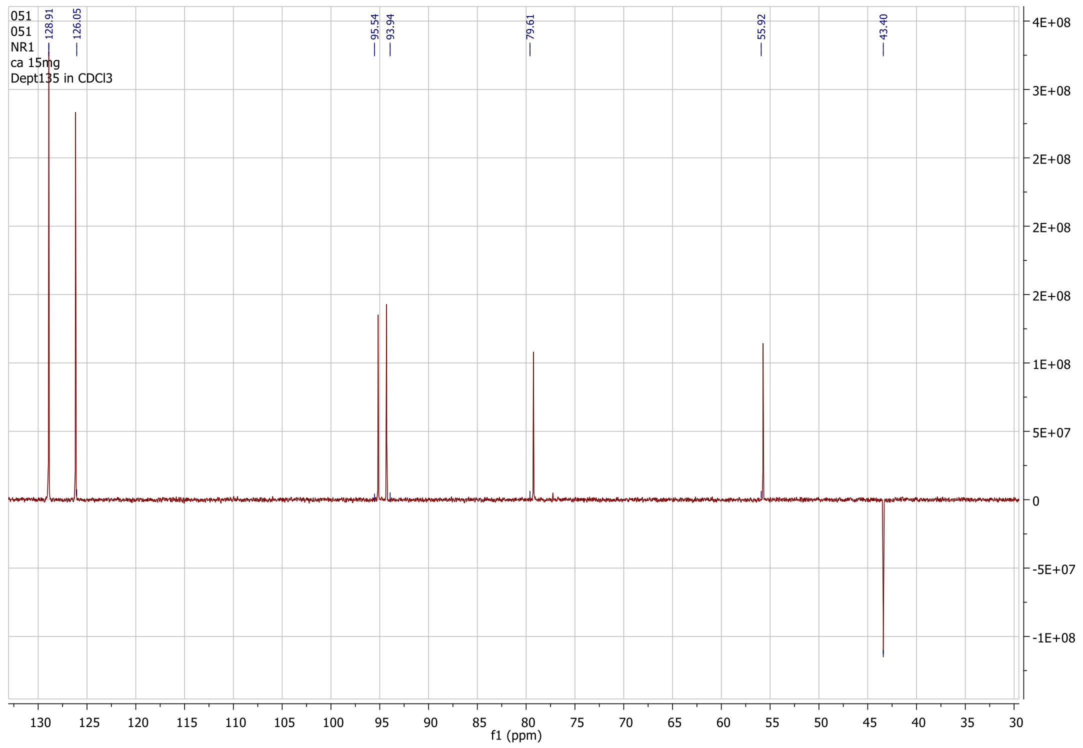

Figure S31. Phase edited HSQC NMR spectrum (300 and 150.09 MHz) of Compound 5, peak 49, 5-hydroxy-7-methoxyflavanone (pinostrobin).

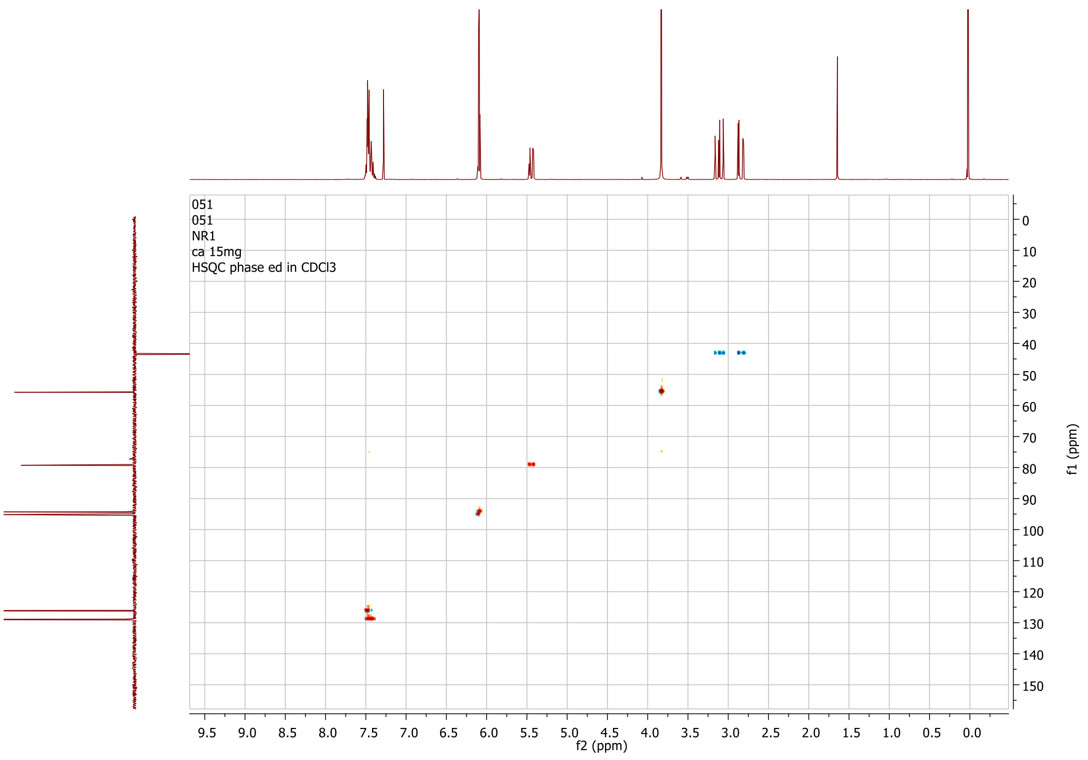

Figure S32. Phase edited HMBC NMR spectrum (300 and 150.09 MHz) of Compound 5, peak 49, 5-hydroxy-7-methoxyflavanone (pinostrobin).

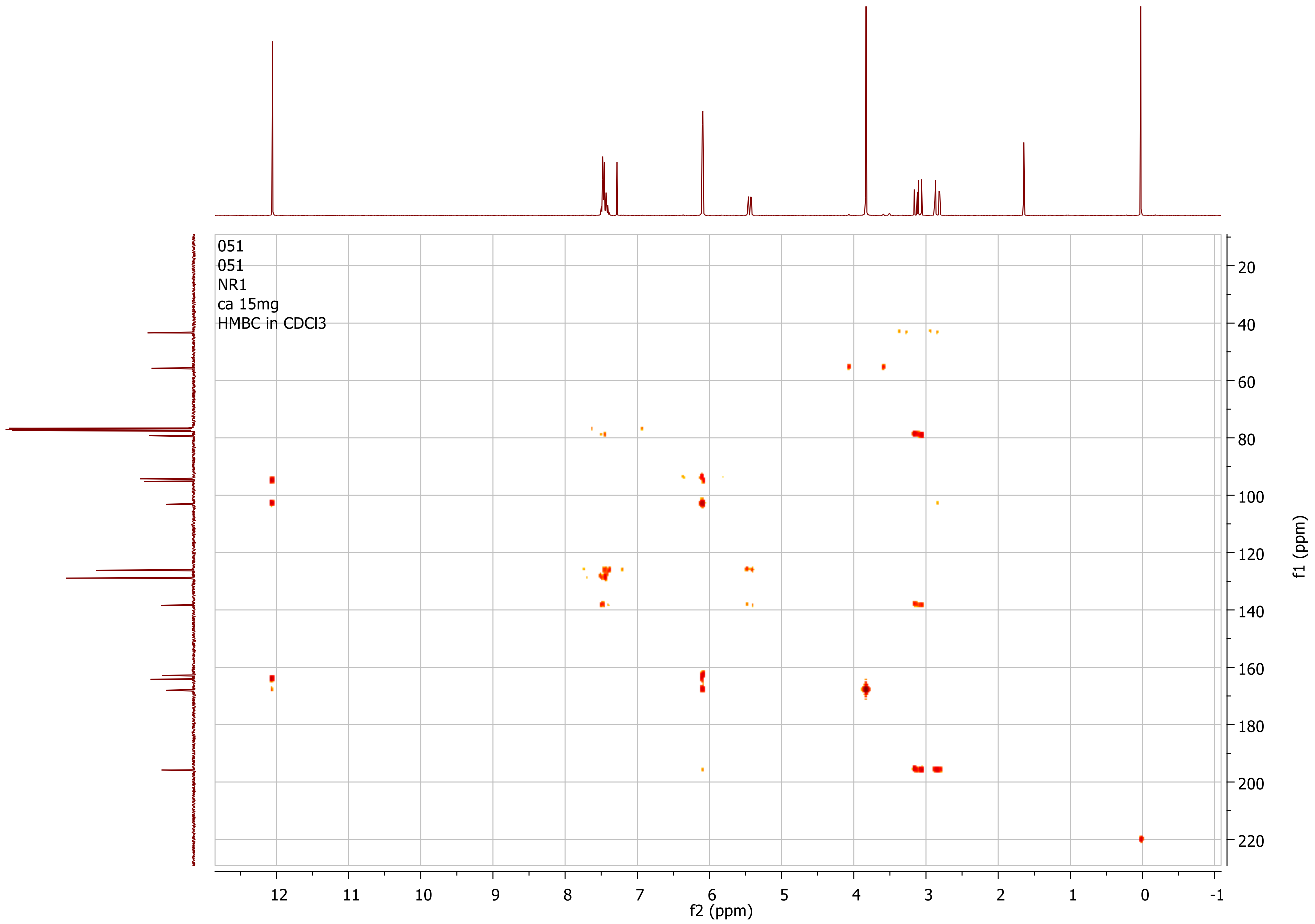

Supplement: Supplementary file 1 [file molecules-25-00520-s001.pdf]
